# Prevalence of functional disorders across Europe: a systematic review and meta-analysis

**DOI:** 10.1007/s10654-024-01109-5

**Published:** 2024-03-29

**Authors:** Caroline Rometsch, Giovanni Mansueto, Frederic Maas Genannt Bermpohl, Alexandra Martin, Fiammetta Cosci

**Affiliations:** 1 Department of Experimental and Clinical Medicine, University of Florence, Largo Brambilla, 3, 50134 Florence, Italy; 2 Department of Health Sciences, University of Florence, Florence, Italy; 3 Department of Psychology, Sigmund Freud University, Milan, Italy; 4 School of Human and Social Sciences, University of Wuppertal, Wuppertal, Germany; 5 Department of Psychiatry and Neuropsychology, Maastricht University, Maastricht, The Netherlands

**Keywords:** Functional disorders, Epidemiology, Prevalence, Meta-analysis, Review

## Abstract

Functional Disorders (FD) refer to persistent somatic symptoms caused by changes in the functioning of bodily processes. Previous findings suggest that FD are highly prevalent, but overall prevalence rates for FD in European countries are scarce. Therefore, the aim of the present work was to estimate the point prevalence of FD in adult general populations. PubMed and Web of Science were searched from inception to June 2022. A generalized linear mixed-effects model for statistical aggregation was used for statistical analyses. A standardized quality assessment was performed, and PRISMA guidelines were followed. A total of 136 studies were included and systematically synthesized resulting in 8 FD diagnoses. The large majority of studies was conducted in the Northern Europe, Spain, and Italy. The overall point prevalence for FD was 8.78% (95% CI from 7.61 to 10.10%) across Europe, with the highest overall point prevalence in Norway (17.68%, 95% CI from 9.56 to 30.38%) and the lowest in Denmark (3.68%, 95% CI from 2.08 to 6.43%). Overall point prevalence rates for specific FD diagnoses resulted in 20.27% (95% CI from 16.51 to 24.63%) for chronic pain, 9.08% (95% CI from 7.31 to 11.22%) for irritable bowel syndrome, and 8.45% (95% CI from 5.40 to 12.97%) for chronic widespread pain. FD are highly prevalent across Europe, which is in line with data worldwide. Rates implicate the need to set priorities to ensure adequate diagnosis and care paths to FD patients by care givers and policy makers.

**Supplementary Information:**

The online version contains supplementary material available at 10.1007/s10654-024-01109-5.

## Introduction

Functional disorders (FD), characterized by persistent (somatic) symptoms such as fatigue, dizziness, bowel symptoms, or neurological dysfunctions, are highly prevalent in all medical settings [1]. An overlap of multiple symptoms in patients is associated with an impaired health status [2–4]. Patients with FD suffer from a reduced quality of life [5], high work disability and illness worries [6]. Furthermore, they cause an increase of health care costs [7] compared to the general population [6].

FD were originally subsumed under the chapter of hysteria and later defined by the absence of organic explanations. Nowadays the role of psychological factors in their onset, worsening, or maintenance is recognized [8]. Functional somatic symptoms have been labeled as “medically unexplained symptoms”, which use has been criticized [9]. The latter clusters chronic somatic symptoms without reproducibly observable pathophysiological mechanisms [10]. The current taxonomy for mental disorders, the Diagnostic and Statistical Manual of Mental Disorders—DSM [11], subsumes FD under *Somatic Symptom Disorder* with the attempt to emphasize positive symptoms such as somatic symptoms with abnormal thoughts, feelings, and behaviors in regard to those symptoms [12]. The commonly used taxonomy of the International Classification of Diseases—ICD [13]—includes FDs under different categories, for instance the rubric of mental disorders with (un-) differentiated somatoform disorders, chronic pain (CP), irritable bowel syndrome (IBS), chronic fatigue syndrome (CFS), or fibromyalgia (FM). There is an ongoing discussion about the criteria for diagnosis, as shown by the introduction of the *Somatic Symptom Disorders* (SSD) of the DSM-5 [12] and the *Bodily Distress Disorders* of the ICD-11 beta draft classification [14], which both led to a controversy about the capture of different dimensions. On the one hand specific features such as distress or excessive thoughts and behaviors have to be present, on the other hand the definitions strive for an absence of features like for instance physical or medical causes [15]. Another diagnostic system, the Diagnostic Criteria for Psychosomatic Research (DCPR), includes FD under the chapter of *persistent somatization, conversion symptom*, or *somatic symptoms secondary to a psychiatric disorder* [16, 17]. New concepts, such as the bodily distress syndrome, were developed [18] and demonstrated to be useful [19].

Discussions regarding diagnostic criteria are open for some FD labels (e.g., IBS [20, 21], CP [22]). For instance, Manning introduced diagnostic criteria for IBS in 1978 [23] while the Rome Foundation published [20] the Rome criteria in 1989. Within each diagnostic system update [24, 25], major changes were introduced. Manning modified the number of symptoms needed for the diagnosis of IBS and in Rome’ revisions defecation patterns were added [25]. A higher sensitivity and accuracy was observed for Manning when compared to Rome criteria [26] but Rome-revision IV became the gold-standard for diagnosing [27]. Similarly, CP diagnostic criteria were defined based on DSM, ICD, or the International Association for the Study of Pain (IASP) systems, even though they differed regarding the time criterion. Indeed, the majority of currently available studies refers to a 6-month duration of pain while a minority refers to a 3-month duration (i.e., those using DSM-5, and ICD-11). For FM, there is, in contrast, a high scientific consensus [28, 29].

Due to this inconsistent use in nosography, point prevalence for FD varies widely, also when applied to specific diagnosis (e.g., CP, IBS, CFS). A lack of reviews summarizing the European epidemiological data of FD is also evident. Hence, the present work has the aim to fill in this gap by systematically reviewing the literature on prevalence of functional disorders in the adult general population across Europe. Since distinct FD are widely overlapping in the general population [30, 31], an overall point prevalence of FD is presented. Additionally, an overall point prevalence of specific diagnoses according to the common nosology and an overall point prevalence in regard to the European country as well as both for specific disorder and country are estimated.

## Methods

### Eligibility criteria

English articles published in peer-reviewed journals on prevalence of FD in European adults (i.e., ≥ 18 years of age) were included. The outcome had to refer to FD point prevalence [32] diagnosed according to the DSM, ICD, DCPR, or standardized criteria (e.g., Manning, Rome, American College of Rheumatology [ACR]) also via self-developed questionnaires if referring to specific standardized criteria. Additional inclusion criteria were: observational design (e.g., cross-sectional, longitudinal, cohort, case–control); general population; sample size of at least 500 subjects, to minimize under- or over-estimation of prevalence and to ensure the inclusion of high-quality research [33, 34] and guarantee statistical robustness [35, 36]. Sex-specific populations were accepted for the systematic review, but were not included in meta-analyses. Studies focusing on special populations (e.g., veterans, students), qualitative studies, and randomized controlled trials were excluded.

### Information sources and search strategy

A systematic search in PubMed and Web of Science was conducted from inception to June 2022. Search terms were any term of FD (for details see Table S2, online supplementary material) combined using the Boolean ‘AND’ operator with ‘Prevalence*’ ‘OR’ and ‘Epidemiol*’. The full search strategy for PubMed is presented in Table S2 (online supplementary material). A manual search of reference lists and a targeted search of grey literature was performed. The review process was streamlined by using the open source online tool Rayyan [37]. The Preferred Reporting Items for Systematic Reviews and Meta-analysis (PRISMA) guidelines [38] were followed. Endnote [39] was used to remove duplicates. Authors were contacted to provide their reports if the full text could not be retrieved. Two reviewers (CR and GM) independently screened potential eligible articles and full-texts, and a third reviewer (FC) was included in case of disagreements. The protocol was preregistered on PROSPERO no. CRD42022298974) [40], as well as on OSF (https://osf.io/w52jm).

### Data extraction and quality assessment

A standardized data extraction form was developed to collect relevant data: reference, population, sample size, study design, diagnostic procedure (diagnostic instrument, additional clinical interview), prevalence estimates. After data extraction, studies were grouped according to specific diagnosis (e.g., IBS, CP, CFS). The methodological quality of studies was verified independently by CR and GM via the Joanna Biggs Institutes’ Critical Appraisal Checklist for Studies Reporting Prevalence Data (JBI) [41]. The JBI [41] assesses study quality via nine items to explore: study participants, sample size, sample power, methods, measurement, statistical analysis, response rate [41]. It allows to collect information based on a 4-point Likert scale (“*yes, no, unclear, not applicable*”) giving a maximum sum score of 9 [42]. Sum score was converted into percentage; over 66% were considered as low, between 44 and 65% as moderate, < 44% as high risk of bias. The Kappa coefficient statistic for interrater reliability showed a very good outcome with 0.91 [43].

### Statistical analysis

If not available in the paper, the point prevalence was calculated, i.e., (number of diagnosed participants/total sample number) × 100. To ensure robustness in case of multiple prevalence estimates collected over time, the first report obtained in the first assessment was used and no studies overlapped regarding the recruitment of patients. Data were analyzed using the Software R Studio (version 4.3.0) with the R function *metaprop* from R package meta (package version 6.2.1.). Data and R syntax are available on OSF (https://osf.io/w52jm). An overall point prevalence rate for FD was calculated using a generalized linear mixed-effects model (GLMM) [44] which logit-transforms proportions [36]. All studies were included in the overall point prevalence calculation. Thereafter, subgroup analyses for specific diagnoses (i.e., IBS, CP with a 6-month duration, CWP), for specific country (i.e., Denmark, France, Germany, Great Britain, Italy, Netherlands, Norway, Spain, Sweden), and a post-hoc analysis regarding the use of validated questionnaires (validated vs. non-validated) were conducted based on a mixed-effects model [36]. In order to be rigorous, these subgroup analyses were run if there was a minimum of 10 studies per group [36]. Additional post-hoc subgroup analysis was conducted for specific diagnosis in regard to each country if there were at least two studies per diagnosis. A subgroup analysis was conducted for the risk of bias including low and moderate risk of bias studies after JBI-rating. The between-study heterogeneity was explored by calculating *τ*^2^ with a Maximum-likelihood estimator [45] as well as via *I*^2^-, *Q*-statistics, and the prediction intervals [46]. Results are reported with 95% Confidence Intervals (CI) assuming a Clopper-Pearson distribution and displayed using forest plots. To examine symmetry and publication bias, Egger’s test and Peters’ regression test were run and a funnel plot of logit transformed proportions was created. Forecast analyses were conducted using meta-regressions with a mixed-effects model to examine whether the year of publication might predict FD point prevalence.

The current study is part of the innovative training network ETUDE (Encompassing Training in fUnctional Disorders across Europe; https://etude-itn.eu/), ultimately aiming to improve the understanding of mechanisms, diagnosis, treatment and stigmatization of Functional Disorders [47].

## Results

### Results of the systematic review

After removal of duplicates, 74,733 articles from databases and 220 from citation searching were screened for eligibility, among them 707 full-text articles were assessed. A total of 136 papers met the inclusion criteria (see Fig. S1, online supplementary material) with 199 point prevalence rates referring to 8 FD diagnoses: headaches (*n* = 3), CFS (*n* = 7), somatization (n = 8), FM (*n* = 11), CWP (*n* = 18), IBS (*n* = 35), CP (*n* = 24), and functional gastrointestinal, neurological, psychiatric symptoms (*n* = 30) (see Table S1, online supplementary material).

### Point prevalence estimates of FD

In total, 199 point prevalence estimates were found ranging from 0.03% for CFS [48] to 62.5% in females with IBS [49].

### Headaches

Three studies on tension type headaches [50–53] reported rates of 13.3% [51], 18.7% [52], and 34% [53], respectively. Kristiansen et al. [52] and Göbel et al. [51] used Norway registers of selected counties while Sjaastad et al. [53] analyzed a rural general population.

### ﻿﻿Chronic fatigue syndrome/Myalgic encephalomyelitis

Seven studies reported prevalence rates up to 8.1% [30, 64–69]. Variations in prevalence rates were found due to the definition of CFS with the higher rates (0.19%) applying the Centers for Disease Control criteria and the lowest rates (0.03%) applying the Epidemiological Case Definition [48].

### Somatization

Eight studies [54–61] reported point prevalence from 0.6 [61] to 35.9% [62]. Overall the studies applied different diagnostic criteria: DSM [54, 56, 57, 59, 60, 63], ICD [61, 62], both ICD/DSM [58]. Among them, Grabe et al. [55] estimated the point prevalence using the DSM-IV with 1.3% for specific somatoform disorder and 19.7% for undifferentiated somatoform disorder.

### Fibromyalgia

Eleven studies [30, 68, 70–81] reported rates between 0.66 [72] and 4.6% [30]. Two studies that used all-female samples had higher rates (10.5%, [71]; 13.5%, [75]). Twelve studies [71–81] applied the ACR 1990 criteria for diagnosing FM showing a prevalence with a range from 0.66 [72] to 13.5% [75]. Mäkelä et al. [70] used the Yunus criteria with prevalence estimates of 0.75%, while Janssens et al. [68] used DSM-IV and ICD-10 resulting a point prevalence of 3%. In some cases, the authors conducted a clinical examination only in a subgroup of the sample and the prevalence rate was calculated on this subgroup. This diagnostic procedure was applied in several studies [71–73, 75, 76, 78, 79] with prevalence rates around 0.75% and 2.4%. The highest prevalence estimates (i.e., 3% and 4.6%) were found when no clinical examinations were conducted [68, 69].

### Chronic widespread pain

Eighteen studies [3, 30, 66, 73, 82–95] provided information on prevalence showing a range from 1.42% [95] up to 20.8% in an all-female twin sample [93]. Applying ACR-1990 criteria [3, 30, 66, 73, 82, 85–90, 92–94, 96], prevalence rates were higher (up to 20.8% [93]) than referring to other criteria (e.g., DSM-IV: 6.7% [91], Manchester: 4.7% [83], others: 1.42% [95]).

### Irritable bowel syndrome

Point prevalence estimates ranged from 2.1% [97] up to 62.5% [49] with a broader IBS-definition in an all-female sample. Heterogeneity in prevalence seems related to the classification system used (e.g., Manning or Rome criteria) and the procedure applied for the assessment. When Rome I criteria were used [97–103], rates were lower than when Manning was used [97, 100]. When Rome II criteria were applied, prevalence was lower than when Manning or Rome I was applied [98]. When Rome IV was used, rates were lower than with Rome III [104].

When a clinical interview was proposed next to self-administered questionnaires [97, 98, 105–107], point prevalence was lower [49, 97, 98, 105–107] than when the clinical interview was not conducted [104, 108].

### Chronic pain

In 12 studies [109–120], pain was measured independently of the body region. Rates ranged from 14.3 [110] to 40% [109, 114]. The range might be this wide due to the heterogeneity of pain regions that were examined. Six studies reported on low back pain with a prevalence of 10–27% [91, 121–126], 4 studies reported on musculoskeletal pain (23.9–45% [127–130]), 3 on pelvic pain (17–26.8% [131–133]), 3 on neck pain (9–22%, [134–136]), 1 on chest pain (44.7% [137]), and 1 on abdominal pain (22.6% [138]).

### Functional gastrointestinal, neurological or psychiatric symptoms

Thirty studies derived through a miscellaneous group of functional gastrointestinal, neurological or psychiatric symptoms. They are described in Table S1 (online supplementary material).

### 3.11. Results of the meta-analyses and subgroup analyses

#### Overall point prevalence

The meta-analyses of 199 estimates including 2.448.164 observations resulted in an overall point prevalence of 8.78% (95% CI from 7.61 to 10.10%) (see online supplementary material Fig. S2). A significant heterogeneity was found (I^2^ = 99.9%, prediction interval [0.1; 0.46]) as well as asymmetry in the funnel plot, Egger’s test (t(197) =  − 10.14, *p* < 0.001) and Peters’ regression test (t(197) = − 4.82, *p* < 0.001) (see Figs. S1 and S2, online supplementary material).

#### Overall point prevalence for specific diagnoses

**Fig. 1:**
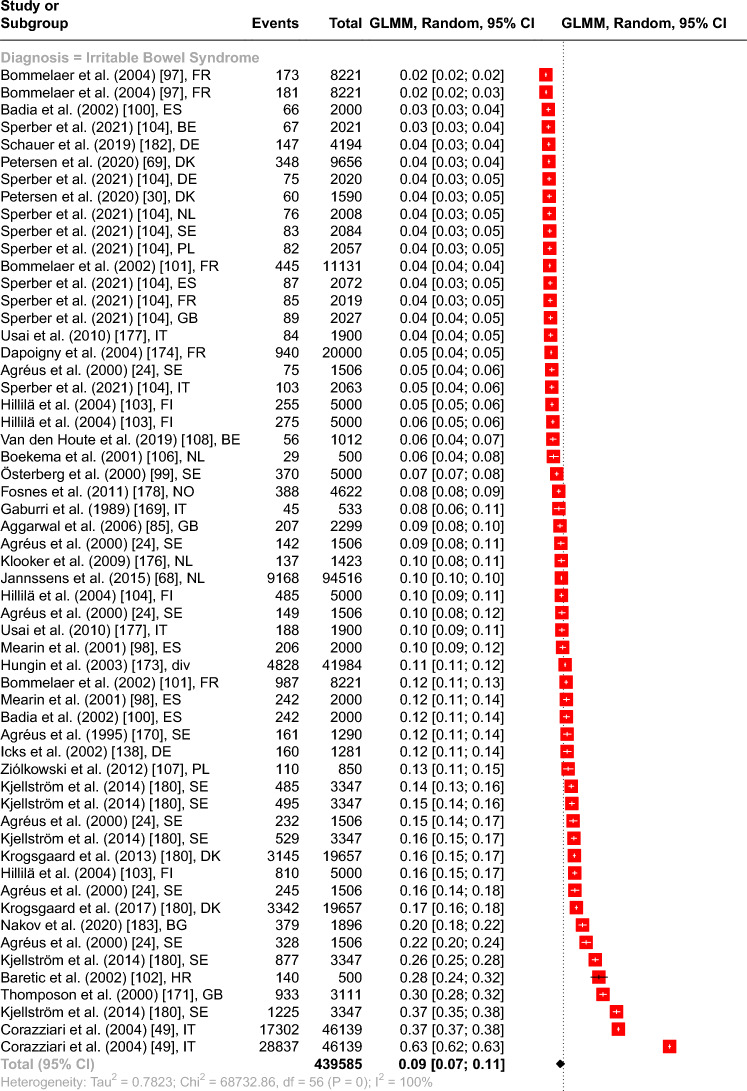

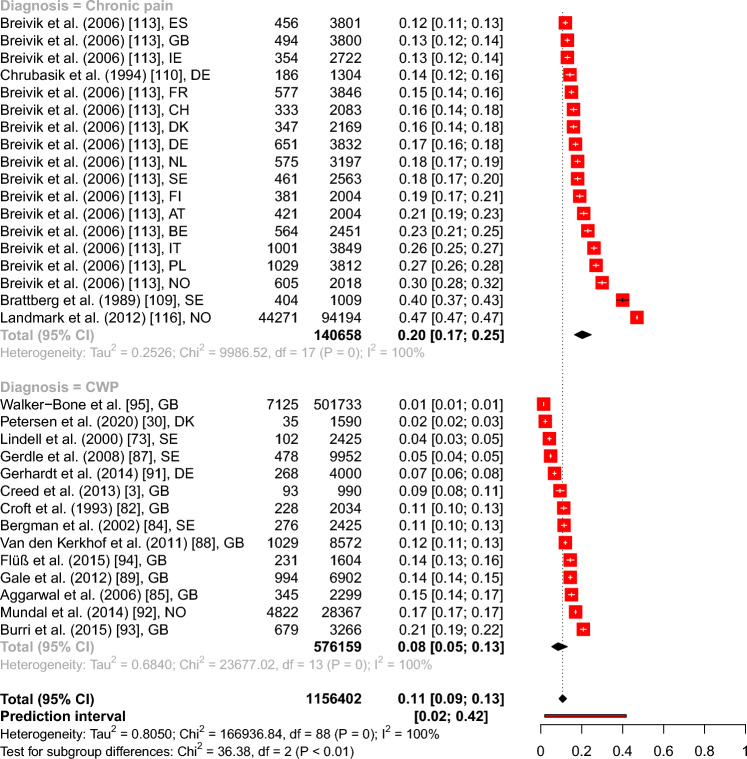
Forest plot of the overall point prevalence rates for IBS, CP, and CWP with 95% confidence intervals and prediction intervals in regard to the author, year of publication, and country. *Note.* *AT* Austria, *BE* Belgium, *BG* Bulgaria, *CH* Switzerland, *DE* Germany, *DK* Denmark, *ES* Spain, *FI* Finland, *FR* France, *GB* Great Britain, *HR* Croatia, *IE* Ireland, *IT* Italy, *NL* The Netherlands, *NO* Norway, *PL* Poland, *SE* Sweden

The overall point prevalence for CP resulted in 20.27% (95% CI from 16.51 to 24.63%), for IBS in 9.08% (95% CI from 7.31 to 11.22%), and for CWP in 8.45% (95% CI from 5.40 to 12.97%). The subgroup analysis of IBS, CP, and CWP included 89 prevalence estimates with 1.156.402 subjects and resulted in a significant between groups difference with *Q*(2) = 36.38, *p* < 0.001 (see Fig. 1).

#### Overall point prevalence per country

**Fig. 2:**
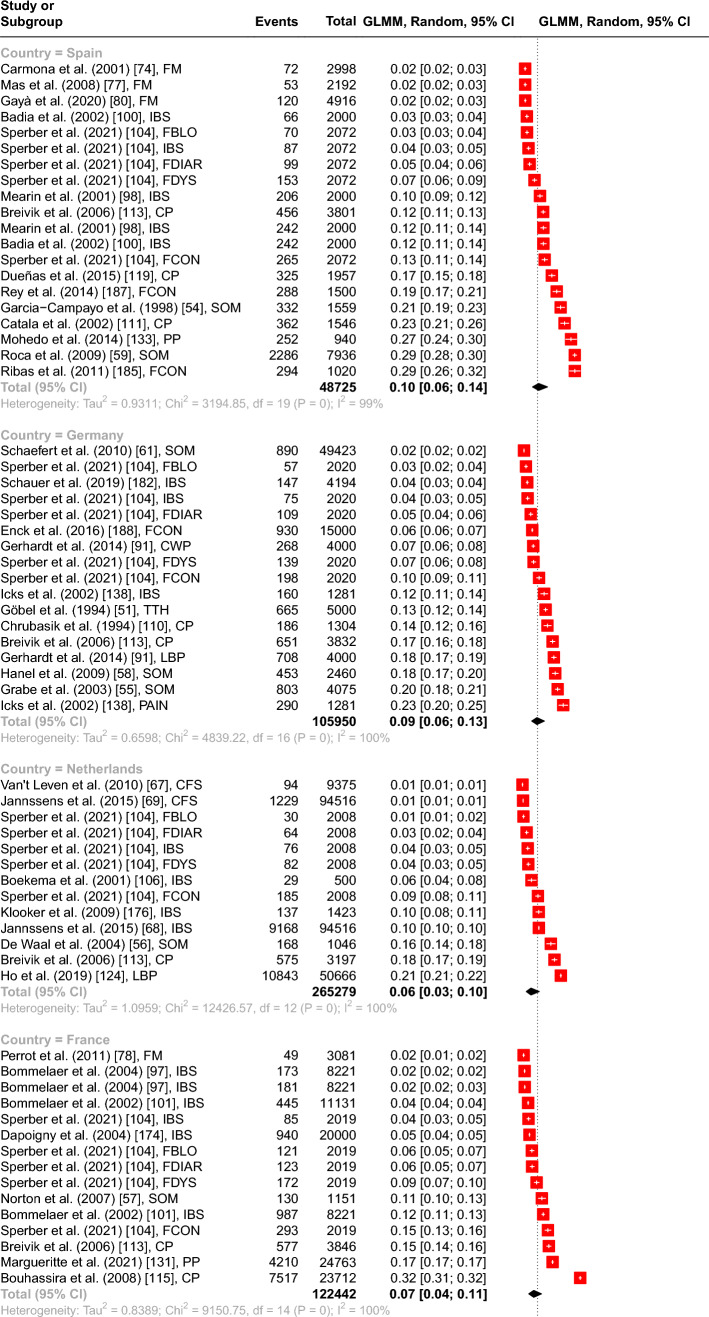

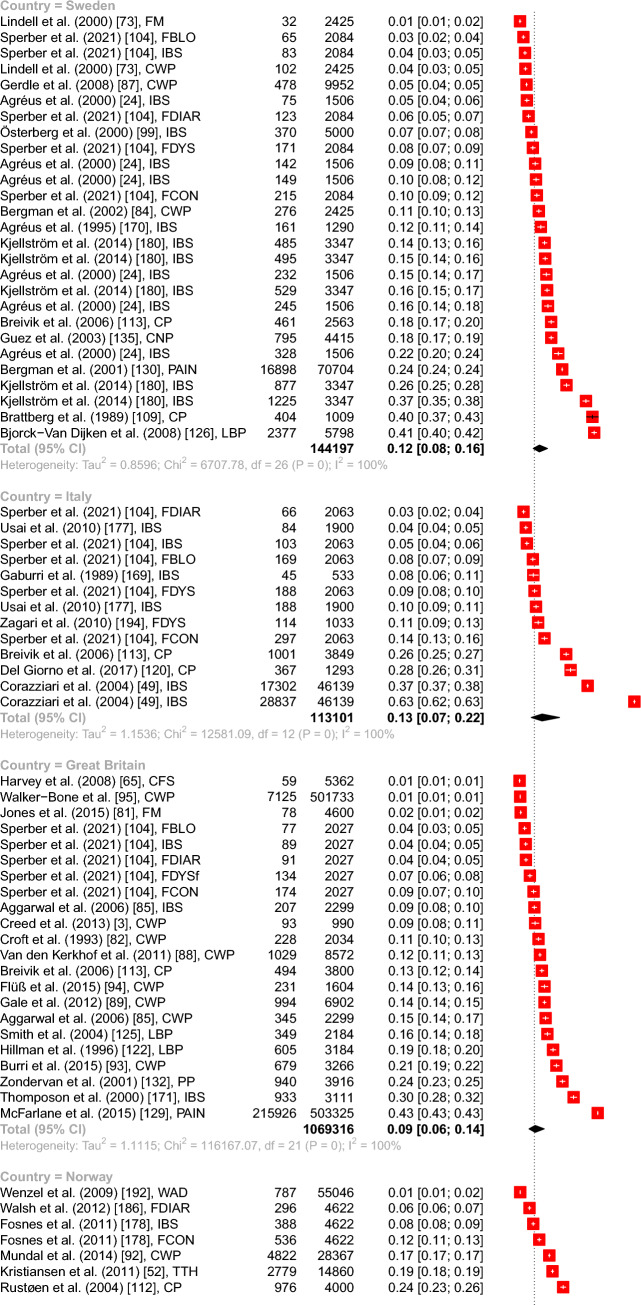

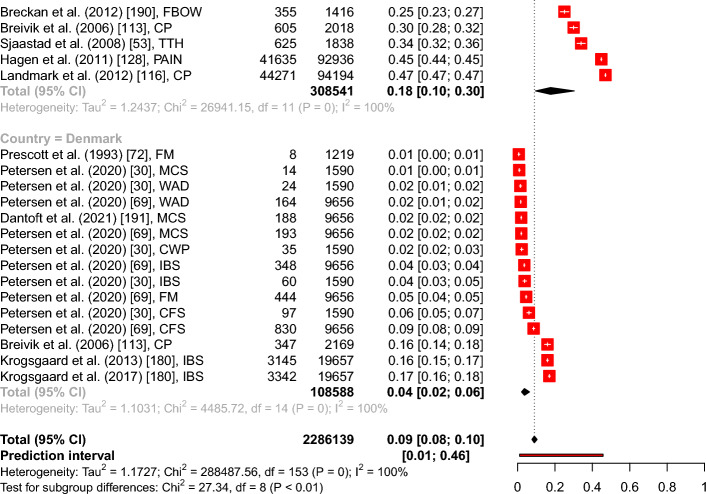
Forest plot of the overall point prevalence of function disorders in regard to the country (with a number ≥ 10 studies per country) with 95% confidence intervals and prediction intervals in regard to the author, year of publication, and specific diagnosis. *Note.* *CP* Chronic pain, *CFS* Chronic fatigue syndrome, *CWP* Chronic widespread pain, *FBLO* Functional bloating, *FBOW* Functional bowel symptoms, *FCON* Functional constipation, *FDIAR* Functional diarrhea, *FDYS* Functional dyspepsia, *FM* Fibromyalgia, *IBS* Irritable Bowel Syndrome, *LBP* Low back pain, *PAIN* musculoskeletal pain, *PP* Pelvic pain, *SOM* Somatization, *TTH* Tension type headaches, *WAD* Whiplash associated disorder

The meta-analyses showed the highest overall point prevalence of FD in Norway with 17.68% (95% CI from 9.56 to 30.38%) and the lowest in Denmark with 3.68% (95% CI from 2.08 to 6.43%). Most studies were conducted in Sweden (*N* = 27), followed by Great Britain (*N* = 22), Spain (*N* = 20), Germany (*N* = 17), France and Denmark (each *N* = 15), Italy, Netherlands (each *N* = 13), and Norway (*N* = 12). The subgroup analysis showed a significant between groups difference with *Q*(8) = 27.34, *p* < 0.001, including 154 prevalence estimates and a total of 2.290.761 observations (see Fig. 2).

#### Overall point prevalence for specific diagnosis regarding each country

The overall point prevalence for specific diagnosis varied according to the country. For IBS, the overall point prevalence was in France 4.09% (95% CI from 2.18 to 7.52%), in Germany 5.51% (95% CI from 1.19 to 22.01%), in Spain 7.42% (95% CI from 3.67 to 14.45%), in the Netherlands 6.83% (95% CI from 3.56 to 12.68%), in Poland 7.26% (95% CI from 0.03 to 96.05%), in Denmark 8.03% (95% CI from 2.30 to 24.44%), in Finland 8.24% (95% CI from 3.79 to 17.02%), in Great Britain 11.12% (95% CI from 1.23 to 55.69%), in Sweden 13.13% (95% CI from 9.26 to 18.31%), and in Italy 14.58% (95% CI from 4.08 to 44.66%). The overall point prevalence for CP resulted in 15.85% in Germany (95% CI from 6.90 to 32.38%), 16.80% in Spain (95% CI from 8.16 to 31.47%), 22.28% in France (95% CI from 0.37 to 95.60%), 26.67% in Italy (95% CI from 16.61 to 39.91%), 27.64% in Sweden (95% CI from 0.26 to 98.27%), and 33.22% in Norway (95% CI from 14.68 to 58.99%). The overall point prevalence of CWP was in Sweden 6.17% (95% CI from 1.97 to 17.73%) and in Great Britain 10.41% (95% CI from 5.47 to 18.92%). The analysis showed an overall prevalence for FM of 1.81% in Denmark (95% CI from 0.00 to 99.42%) and of 2.42% (95% CI from 1.85 to 3.18%) in Spain. In Germany, the overall prevalence of somatization was 9.13% (95% CI from 0.50 to 66.65%) and in Spain was 25% (95% CI from 5.17 to 67.09%). The overall prevalence of CFS was 1.19% in the Netherlands (95% CI from 0.36 to 3.83%) and 7.41% in Denmark (95% CI from 1.48 to 29.84%). For more details see the online supplementary material (see Fig. S3 online supplementary material).

#### Overall point prevalence according to validation or non-validation of tools used

Seventy-nine studies used a validated tool with an overall point prevalence of 10.19% (95% CI from 8.17 to 12.64%) while 119 studies used non-validated tools with an overall point prevalence of 7.85% (95% CI from 6.51 to 9.44%). Non-significant effects was found between groups (*Q*(1) = 3.25, *p* = 0.071).

### Forecast analyses

Using the prevalence data with the year of publication, findings indicate a significant yearly decrease of 3.58 (95% CI − 5.43%; − 1.73%) for FD point prevalence.

### Risk of bias analysis

For the meta-analysis on point-prevalence, 166 studies showed a low risk of bias with an overall point prevalence of 8.01 (95% CI from 6.83 to 9.38%), and 33 a moderate risk of bias with an overall point prevalence of 13.66 (95% CI from 10.31 to 17.90%). The subgroup-analysis resulted in a significant between groups effect (*Q*(1) = 11.02, *p* < 0.001). Studies with high risk of bias were not included in the meta-analysis.

## Discussion

The present systematic review and meta-analyses of the literature on FD point prevalence in adult European populations revealed a wide range from 0.66% for FM up to 62.5% for IBS. The meta-analytic aggregation resulted in an overall point prevalence of FD of 8.78% (95% CI from 7.61 to 10.10%). Prevalence rates of FD were highest in Norway with 17.68% (95% CI from 9.56 to 30.38%) and lowest in Denmark with 3.68% (95% CI from 2.08 to 6.43%). The majority of epidemiological studies was found in Northern European countries, Spain, and Italy. The overall point prevalence rate of CP was 20.27% (95% CI from 16.51 to 24.63%), IBS showed a rate of 9.08% (95% CI from 7.31 to 11.22%), and CWP a rate of 8.45% (95% CI from 5.40 to 12.97%).

The distribution of prevalence estimates according to the systematic review was in some cases homogeneous within the same diagnosis (e.g., CFS, FM), in other cases heterogeneous (e.g., somatization, CWP, IBS, CP). This seems to be based in differences in the diagnostic system used (e.g., DSM vs ICD or Manning vs Rome), in the assessment procedures applied (e.g., validated tools vs. non-validated tools, adding a clinical interview), and in the country in which data were collected. To diagnose a specific FD, not only different diagnostic systems were applied (e.g., ICD and DSM for somatization, Manning and Rome for IBS, and ICD, DSM and IASP for CP) but also different revised versions of the system itself were used (e.g., ICHD version 1–4 for headaches, Rome version 1–4 for IBS [139]). To the best of our knowledge, there is a research gap concerning common nosology of FD terms’ impact on epidemiological outcomes. In particular, heterogeneity in prevalence rates for specific FD diagnoses (e.g., headaches, CP, CWP, IBS) implicates that prevalence rates differ in regard to the custom taxonomy and criteria used. Finding the “true” prevalence of FD requires a precise methodological design applying standardized criteria. This might include the assessment methods for diagnosing. For IBS and FM, common assessment methods were applied across the studies reviewed: self-administered questionnaires, (personal or telephone) clinical interviews or examinations, and their combination [216]. Findings of the systematic synthesis resulted in a lower point prevalence when a clinical interview or examination was applied instead of studies using a self-administered questionnaire as diagnostic tool. When validated tools were applied, the overall point prevalence was higher compared to the application of non-validated instruments. Findings are in line with an investigation on Danish adult FD patients, showing higher prevalence rates when a self-report tool was applied in comparison to when clinical interviews were conducted [140]. Wide ranges in prevalence rates were described across several (psycho-)somatic disorders, leading to the conclusion that there is a need for a common scientific practice applying uniform methodological validated assessment tools to ensure comparability of results.

Although there is heterogeneity of results, the overall point prevalence for all FD combined was 8.78% (95% CI from 7.61 to 10.10%). This is the first study to provide a quantitative synthesis of epidemiological results on the general population across Europe. Globally, the prevalence of FD in the general population was estimated at 12.9% (95% CI from 12.5 to 13.3%, applying the SSD criteria) [141]. In the primary care context with a worldwide perspective, epidemiological investigations using metanalytic aggregations revealed slightly lower overall point prevalence rates for the somatization disorder with a range from 0.8% (95% CI 0.3–1.4%, I^2^ = 86%) to 5.9% (95% CI 2.4–9.4%, I^2^ = 96%) and higher overall point prevalence rates from 0.2% (95% CI 0.9–79.4%; I^2^ = 98%) to 49% (95% CI 18–79.8%, I^2^ = 98%) for the term “medically unexplained symptoms” [142] compared to the here calculated results for FD. In specialized health-care systems, FD are even prevalent to higher degrees (from 29% [143] up to 66% [144]). Epidemiological findings differ in regard to the context (general population vs. primary/specialized care context) and the diagnosis (FD vs. specific FD diagnosis), the epidemiological aggregation of this study implicates that one out of ten adults in the general population suffer from FD, concluding that FD are highly prevalent across Europe. This also applies to the overall point prevalence rate of CP which resulted in 20.27% (95% CI from 16.51 to 24.63%). Worldwide, prevalence estimates show a wide range from 8.7 to 64.4% [13], and varies widely according to age of the sample, pain location or body region involved [14]. The overall point prevalence of IBS resulted in 9.08% (95% CI from 7.31 to 11.22%) across Europe, which is consistent with global estimates for IBS with 11.2% (95% CI 9.8–12.8%) [145]. Globally investigated prevalence on IBS varied depending on the country in which the research was conducted (lowest prevalence in Southeast Asia with 7.0% and highest in South America with 21.0%) and the diagnostic criteria applied (highest prevalence when 3 or more of the Manning criteria were used (14.0%; 95% CI 10.0–17.0%), the lowest was found when the Rome I criteria were used (8.8%; 95% CI 6.8–11.2%)) [145]. This serves as an example that prevalence rates become more homogeneous the more consensus exists regarding the custom taxonomy applied. Finally, the overall point prevalence of CWP was 8.45% (95% CI from 5.40 to 12.97%), similarly to CP there is a wide range of prevalence ranging from 1.4 to 24% [146]. 

There are several challenges in diagnosing FD even when clinicians follow one custom taxonomy, since few but impairing symptoms may not be captured, and also the utility of custom taxonomies in primary care is not yet proven evidentially (e.g., in BDS) [18]. To be noted that there is an overlap of functional somatic symptoms among multiple syndromes, such as CWP, IBS and CFS [3]. This may lead to difficulties in clearly distinguishing specific FD diagnoses, which may result in an overestimation in epidemiological investigations. To overcome diagnostic insecurities and imprecise clinical diagnosis, a new classification system for FD was proposed with regard to the body system in which those troublesome symptoms may occur (e.g., musculoskeletal, gastrointestinal, cardio-respiratory, genito-urinary, nervous system or fatigue related). This classification differs between one or more affected organ systems (so-called multi-system or single system) and a single persistent symptom (so-called single symptom) [147]. Psychological or behavioral dysfunctions may be present, but are not necessary for the diagnosis [147]. An occurrence with symptom-congruent medical conditions is possible and probable [147].

Some countries contributed a high number of studies (e.g., Sweden, UK), while others are missing (e.g., Austria, Belgium, Czech Republic, Hungary, Romania, Portugal, Lithuania). More Northern countries (e.g., Denmark, Norway, or Sweden) reported on prevalence taking the advantage of birth registers [148]. In addition, there are some European countries in which psychosomatic medicine is practiced [149] as an independent discipline, which entails having an institutional organization [150]. This may imply that some countries (e.g., Denmark, Germany, Great Britain, Netherlands, Norway, France, Italy, Spain, and Sweden) conduct more epidemiological studies than others, which may cause biases (over- or under-estimation) of the overall point prevalence estimation of FD and specific diagnoses across Europe. The highest overall prevalence rate was observed in Norway and the lowest in Denmark. Considering the prevalence estimates of specific diagnoses at the country level, significant differences were found. This suggests that there is a considerable degree of heterogeneity in the prevalence rates of FD across and within countries. This might echo alterations in methods applied to run the research and might be based on political, cultural, and health systems differences. Going more into details, the within countries difference can be related to the use of different diagnostic criteria (e.g., Rome I/II criteria or the modified Rome criteria according to Manning), to the use of clinical interviews in the assessment procedure, to the geographical location (rural vs urban settings)—might also play a role, and to the population characteristics, such as age, sex, socio-economic status. Additionally, differences in healthcare access and utilization patterns can play a role as well as the statistical methods used for data analysis, particularly in handling missing data. Heterogeneity might also origin from a different understanding of genesis and etiological mechanisms of FD, illness behaviors in differential cultural contexts, prevention approaches, and stigmatization. The health ministry of Denmark developed and implemented a mental health promotion package to regulate the management of mental illness [151], including digital psychiatry, early interventions, civil society initiatives, anti-stigmatization campaigns, suicide prevention [152]. National guidelines to treat FD in Denmark exist [153], which may elucidate an appropriate treatment for patients with FD and the low rate of FD. However, an improvement in the recognition, treatment, and anti-stigmatizing of FD is called for [154], such approaches to further improve care for patients with FD presents the awareness and relevance that FD have on the Danish country level. The Norwegian mental health systems show to be less efficient in treating patients with mental health problems [155] leading to longer periods of sick leave [156]. To the best of our knowledge, national guidelines for FD are lacking in Norway. Guidelines serve the aim to provide a set of structured recommendations for clinicians to confidently diagnose and treat patients to improve quality of care [157]. The World Health Organization’s guidelines in mental disorders [158], also adapted for the primary care setting [159], can support existing and future national and international guidelines developed by disorder-specific organizations.

Patient organizations or initiatives, as for example in the field of functional neurological disorders (e.g., FND Hope for functional neurological disorders [160]), are essential pioneers for the development of national and international guidelines. Unfortunately there are no current initiatives by European Parliament on FD, but they take stand on mental health not least due to the sequelae of the COVID-19 pandemic [161].

Finally, investments have a key role too. Denmark spends the most total costs (direct and indirect, measured by using the gross domestic direct product) in the sector of mental disorders in comparison to other EU countries, followed by Germany, Austria, and Spain [162], which might give another explanation for more engagement on the mental health sector to develop such national guidelines.

The present study has limitations. The PRISMA reporting guidelines [38] were followed to guarantee transparency and accuracy, but the results might not be free from biases. The inclusion of English published papers ensured the synthesis of high-quality studies but may have excluded some written in other languages. The inclusion of studies with a study population of ≥ 500 participants may be another limitation, since studies on smaller samples might use more accurate diagnostic procedures [163]. The Eggers’ test, Peters’ regression test, and the funnel plot indicated asymmetry, which may imply either publication biases or small-study effect, or both. Overall, even though tests of heterogeneity for each prevalence rate and subgroup demonstrated considerable variability, a precise statistical procedure was chosen with a-priori definitions of subgroups to reduce heterogeneity. Furthermore, the JBI revealed the majority of studies had a low risk of bias. However, studies with a low or moderate risk of bias were included in the analysis, which might lead to a bias of the actual results.

In conclusion, findings demonstrate a high prevalence, and thus impact, of FD in and on European populations [164]. Findings are in line with global estimations of the FD prevalence, however, comparability is problematic due to nosography and methodological challenges. Core outcomes are urgently needed to overcome heterogeneity in epidemiological studies on FD, in particular: a generally valid and recognized classification system and methodological assessment throughout Europe. For this, guidelines on national, but especially on international level, would be of immense importance and should straightaway be developed by leading European organizations and networks as a support across Europe.

These epidemiological data represent a basic principle of market research of supply and demand: the higher the demand due to FD patients, the higher the need of a functioning public health system with adequate care paths. Adequate health care should be provided under the light of the WHO practical suggestions of *patient engagement* [165], which highlights the relevance of patients’ active role within the decision-making process [166, 167] and brings patients’ transition from object to subjects in health care [168]. Results can also help to push healthcare policymakers acknowledging the relevance of FD and acting accordingly. This epidemiological estimations are essential to plan public health care efforts, scaling resources and needs for disease-modifying treatments and effective low-cost interventions [169].

### Supplementary Information

Below is the link to the electronic supplementary material.

Supplementary file1 (DOCX 250 kb)
