# Supplementary material for: Prevalence of functional disorders across Europe: a systematic review and meta-analysis
Source: Eur J Epidemiol. 2024 Mar 29;39(6):571–86. doi: 10.1007/s10654-024-01109-5 (PMC11249491; doi:10.1007/s10654-024-01109-5)
Supplement: Supplementary file 1 — Supplementary file1 (DOCX 250 kb) [file 10654_2024_1109_MOESM1_ESM.docx]

**Prevalence of functional disorders across Europe: A systematic review and meta-analysis**

Caroline Rometsch^1^, Giovanni Mansueto^2,3^, Frederic Maas genannt Bermpohl^4^, Alexandra Martin^4^, Fiammetta Cosci^2,5^

^1^ Department of Experimental and Clinical Medicine, University of Florence, Italy

^2^ Department of Health Sciences, University of Florence, Italy

^3^ Department of Psychology, Sigmund Freud University, Milano, Italy

^4^ School of Human and Social Sciences, University of Wuppertal, Wuppertal, Germany

^5^ Department of Psychiatry and Neuropsychology, Maastricht University, Netherlands

**Corresponding author:**

Caroline Rometsch

Department of Experimental and Clinical Medicine

University of Florence

Largo Brambilla, 3

50134 Firenze

Italy

Carolina.Rometsch@unifi.it

ORCID:

Caroline Rometsch: 0000-0002-3172-0823

Fiammetta Cosci: 0000-0002-5022-0488

Frederic Maas genannt Bermpohl: 0000-0002-2370-4946

Alexandra Martin: 0000-0002-4235-8591

**Keywords:**

Functional disorders, epidemiology, prevalence, meta-analysis, review

**Acknowledgments**

This project has received funding from the European Union’s Horizon 2020 research and innovation programme under the Marie Skłodowska Curie grant agreement No 956673.

# Supplementary material

# Table S1. Studies ordered by diagnosis included in the systematic review with rating of the Joanna Biggs Institutes` Critical Appraisal Checklist for Studies Reporting Prevalence Data rating system (JBI)

| Author | Year | Country | Sample size | Gender | Age | Region | Study design | Diagnostic criteria | Diagnostic instrument | Additional clinical interview | Prevalence estimate | JBI |
| --- | --- | --- | --- | --- | --- | --- | --- | --- | --- | --- | --- | --- |
| Results for chronic fatigue syndrome | | | | | | | | | | |  |  |
| Líndal et al. [1] | 2002 | Iceland | 4000 | both | 19–75 | Diverse | Cross-sectional | Australian, British and American | Self-developed questionnaire | no | 1.4% | 5 |
| Harvey et al. [2] | 2008 | UK | 5362 | both | 53 | Diverse | Cross-sectional | Recorded diagnosis by medical caregiver | Present State Examination and Psychiatric Symptom Frequency scale | Yes | 1.1% (95% 0.8 –1.5 | 7 |
| Kato et al. [3] | 2009 | Sweden | 31318 twins | both | 41 – 64 | Diverse | Cross-sectional | ACR 1990 criteria, Fukuda | Self-developed | Yes | m: 3.5%,  w: 8.1% | 7 |
| Van't Leven et al. [4] | 2010 | Netherlands | 9375 | both | ≥18 | Diverse | Cross-sectional | CDC 1994 | SFQ; Rand 36 Physical functioning;  Symptom criteria for CFS according to the CDC-94 | No | 1.0% (95% CI 0.8–1.2%) | 8 |
| Janssens et al. [5] | 2015 | Netherlands | 94516 | both | 25–50 | Diverse | Cross-sectional | DSM-IV and ICD-10 | MINI | yes | 1.3% (n = 1166) | 8 |
| Petersen et al. [6] | 2020 | Denmark | 9656 | both | 18 – 76 | Diverse | Cross-sectional | According to diagnostic system | Self-developed, SF-36 | No | 8.6% (8.1-9.2) | 7 |
| Petersen et al. [7] | 2020 | Denmark | 1590 | both | 44 – 63 | Diverse | Cross-sectional | EURO-SOMA | Self-developed | Yes | 6.1% (5.1–7.2) | 9 |
| Results for Fibromyalgia | | | | | | | | | | |  |  |
| Mäkelä et al. [8] | 1991 | Finland | 3434 | both | ≥30 | Urban | Cross-sectional | Modified Yunus criteria | Modified questionnaire | yes | 0.75% | 7 |
| Forseth et al. [9] | 1992 | Norway | 2498 | only  females | 20-49 | Urban | Cross-sectional | ACR-1990 criteria | Self-developed | yes | 10.5% (95% 6.4-14.6) | 7 |
| Prescott et al. [10] | 1993 | Denmark | 1219 | both | 18-79 | Urban | Cross-sectional | ACR-1990 criteria | Self-developed | yes | 0.66% (95% 0.28%-1.29%) | 8 |
| Lindell et al. [11] | 2000 | Sweden | 2425 | both | 20-74 | Urban | Cross-sectional | ACR-1990 criteria | SF-36 | yes | 1.3% (95% CI 0.8-1.7; n = 2425) | 9 |
| Carmona et al. [12] | 2001 | Spain | 2998 | both | ≥20 | Urban | Cross-sectional | ACR-1990 criteria | SF-12,  HAQ | yes | 2.4% (1.5 to 3.2) | 9 |
| Schochat et al. [13] | 2003 | Germany | 3174 | only females | 35 to 74 | Urban | Cross-sectional | ACR-1990 criteria | FFbH-R,  BL scale | yes | 13.5% | 6 |
| Mas et al. [14] | 2008 | Spain | 2192 | both | ≥20 | Urban/rural | Cross-sectional | ACR-1990 criteria | Internet based:  SF-12, HAQ | no | 2.4% [95% CI: 1.5-3.2] | 8 |
| Perrot et al. [15] | 2011 | France | 3081 | both | ≥18 | Urban | Cross-sectional | ACR-1990 criteria | LFES Questionnaire,  SF36, HADS, stress VAS, Co morbidities and Regional pain score | Yes | 1.6 (CI95: 1.2%; 2.0%) | 9 |
| Lourenço et al. [16] | 2015 | Portugal | 1719 | both | 21 | Urban | Longitudinal | ACR-1990 criteria | SF-36, BDI, FSQ | yes | 1.0% | 9 |
| Jones et al. [17] | 2015 | UK | 4600 | both | ≥25 | Diverse | Cross-sectional | ACR-1990 criteria,  Modified ACR 1990 criteria | Widespread Pain Index of the ACR | yes | 1.7% [95% CI] 0.7–2.8), 1.2% (95% CI 0.3–2.1), and 5.4% (95% CI 4.7–6.1) | 7 |
| Janssens et al. [5] | 2015 | Netherlands | 94516 | both | 25-50 | Diverse | Cross-sectional | DSM-IV and ICD-10 | MINI | yes | 3.0% (n = 2765) | 8 |
| Gayà et al. [18] | 2020 | Spain | 4916 | both | ≥20 | Diverse | Cross-sectional | ACR-1990 criteria | Self-developed interview questionnaire | Yes | 2.45% (95% CI, 2.06-2.90) | 7 |
| Petersen et al. [6] | 2020 | Denmark | 9656 | both | 18 – 76 | Diverse | Cross-sectional | According to diagnostic system | Self-developed, SF-36 | No | 4.6% (4.2-5.1) | 7 |
| Results for Chronic widespread pain | | | | | | | | | | |  |  |
| Croft et al. [19] | 1993 | UK | 2034 | both | 18-85 | Diverse | Cross-sectional | ACR-1990 criteria | Self-developed |  | 11.2% | 7 |
| Lindell et al. [11] | 2000 | Sweden | 2425 | both | 20-74 | Urban | Cross-sectional | ACR-1990 criteria | Self-developed, SF-36 | yes | 4.2% (95% CI 3.4-5.0) | 9 |
| Bergman et al. [20] | 2002 | Sweden | 2425 | both | 20 – 74 | Urban | Longitudinal cohort | ACR-1990 criteria | Self-developed, SF-36 | No | 1995: 11.4%(10.1-12.6),  1998: 11.2(9.8-12.7) | 8 |
| Aggarwal et al. [21] | 2006 | UK | 2299 | both | 18 – 75 | Urban | Cross-sectional | ACR-1990 criteria | Self-developed, HADS, Health anxiety questionnaire, Sleep problem scale, Somatic symptom checklist | No | 15% | 7 |
| Gerdle et al. [22] | 2008 | Sweden | 9952 | both | 18 – 74 | Diverse | Cross-sectional | ACR-1990 criteria, Manchester | Self-developed | No | ACR: 4.8%,  Manchester: 7.4% | 7 |
| Kato et al. [3] | 2009 | Sweden | 31318 twins | both | 41 – 64 | Diverse | Cross-sectional | ACR-1990 criteria | Self-developed | Yes | m: 2.0%,  w: 6.8% | 7 |
| Van den Kerkhof et al. [23] | 2011 | UK | 8572 | both | 45 years | Diverse | Cross-sectional | ACR-1990 criteria | Self-developed | No | 12.0% | 7 |
| Gale et al. [24] | 2012 | UK | 6902 | both | 45 years | Diverse | Longitudinal | ACR-1990 criteria | Self-developed | No | 14.4% | 7 |
| Creed et al. [25] | 2013 | UK | 990 | both | 25 – 65 | Urban | Cross-sectional | ACR-1990 criteria | Self-developed, HADS | No | 9.4% | 6 |
| Gerhardt et al. [26] | 2014 | Germany | 4000 | both | ≥18 | Diverse | Longitudinal | Pain duration of 3 months,  DSM-IV | Self-developed, SF-36. PHQ-D | No | 6.7% | 7 |
| Mundal et al. [27] | 2014 | Norway | 28367 | both | ≥20 | Diverse | Longitudinal | ACR-1990 criteria | Self-developed,  Standardized Nordic Questionnaire (SNQ) | Yes | 17%, | 8 |
| Burri et al. [28] | 2015 | UK | 3266 twins | women | 18 – 89 | Diverse | Cross-sectional | ACR-1990 criteria | Self-developed, LFESSQ,  CIDI | No | 20.8% | 7 |
| Flüß et al. [29] | 2015 | Scotland | 1604 | both | ≥25 | Urban | Cross sectional | ACR-1990 criteria | Self-developed | No | 14.4% | 6 |
| Walker-Bone et al. [30] | 2016 | UK | 501733 | both | 40 – 69 | Diverse | Cross-sectional | Pain duration of 3 months | Self-developed | No | 1.42% | 6 |
| Petersen et al. [7] | 2020 | Denmark | 1590 | both | 44 – 63 | Diverse | Cross-sectional | ACR-1990 criteria | Self-developed | Yes | 2.2% (1.8–2.8) | 9 |
| Results of tension type headaches | | | | | | | | | | | |  |

| Göbel et al. [31] | 1994 | Germany | 5000 | both | ≥18 | Diverse | Cross-sectional | IHS-1 | Kiel headache questionnaire | No | 13.3% | 6 |
| --- | --- | --- | --- | --- | --- | --- | --- | --- | --- | --- | --- | --- |
| Sjaastad et al. [32] | 2008 | Norway | 1838 | both | 18 – 65 | Rural | Cross-sectional | IHS-l | Self-developed | Yes | 34% | 5 |
| Kristiansen et al. [33] | 2011 | Norway | 14860 | both | 20 – 80 | Diverse | Cross-sectional | ICHD-2 | Self-developed, HADS, Berlin Questionnaire | Yes | 18.7% | 8 |
| Somatization | | | | | | | | | | | | |
| Kato et al. [3] | 2009 | Sweden | 31318 twins | both | 41 – 64 | Diverse | Cross-sectional | Defintion | Self-developed | Yes | m: 17.8%,  w: 32.6%, | 7 |
| Garcia-Campayo et al. [34] | 1998 | Spain | 1559 | both | ≥20 | Urban | Cross-sectional | DSM-lll, ICD-10 | GHQ-28, CAGE, MMSE, SPPI | Yes | 21.3% | 5 |
| Grabe et al. [35] | 2003 | Germany | 4075 | both | 18 – 64 | Urban | Cross-sectional | DSM-IV | Composite International Diagnostic Interview | Yes | Unidfferentiated somatoform disorder: 19.7%,  Specific somatoform disorder: 1.3% | 8 |
| De Waal et al. [36] | 2004 | Netherlands | 1046 | both | 25 – 80 | Urban | Cross-sectional | DSM-lll | SF-36, HADS, PSC | Yes | 16.1% (95% CI12.8-19.4 | 9 |
| Norton et al. [37] | 2007 | France | 1151 | both | ≥18 | Urban | Cross-sectional | DSM-IV | CSRI, PHQ, PRIME-MD | Yes | 11.3% | 7 |
| Hanel et al. [38] | 2009 | Germany | 2460 | both | 18 – 65 | Diverse | Cross-sectional | ICD-11, DSM-V | PHQ-15, PHQ-9, WI-7, DDPRQ-10, | Yes | 18.4% | 7 |
| Roca et al. [39] | 2009 | Spain | 7936 | both | ≥18 | Urban | Cross-sectional | DSM-IV | PRIME-MD | Yes | 28.8% (27.8–29.8) | 7 |
| Schaefert et al. [40] | 2010 | Germany | 49423 | both | ≥20 | Diverse | Cross-sectional | ICD-10 | ICPC-2-E | Yes | P75: 0.6%, F43.3-9: 1.8% | 7 |
| Haftgoli et al. [41] | 2010 | Switzerland | 917 | both | ≥18 | Urban | Cross-sectional | DSM-IV | PHQ, PRIME-MD | Yes | 15.1% (95% CI = 12.8% to 17.5%) | 8 |
| Results for irritable bowel syndrome | | | | | | | | | | | | |
| Gaburri et al. [42] | 1989 | Italy | 533 | both | ≥18 | Urban | Cross-sectional | Clinical | Self-developed | Yes | 8.5% | 5 |
| Heaton et al. [43] | 1992 | UK | 1896 | both | ≥25 | Urban | Cross-sectional | Manning | Self-developed | Yes | m: 5%,  w: 13% | 7 |
| Agréus et al. [44] | 1995 | Sweden | 1290 | both | 20 – 79 | Diverse | Cross-sectional | Self-developed | Self-developed | No | 12.5% | 6 |
| Agréus et al. [45] | 2000 | Sweden | 1506 | both | 20 – 87 | Rural | Cross-sectional | modified Rome criteria Manning,  Swedish criteria | ASQ, BDQ | No | 16.3% (11.2-24.4), 21.8% (16.1-27.5), 15.4% (10.4-20.4);  5.0% (2.0-8.0), 9.9%(5.8-14.0). 9.4% (5.4-13.4) | 7 |
| Thompson et al. [46] | 2000 | UK | 3111 | both | 18 – 89 | Urban | Cross-sectional | Manning,  Rome | N/A | Yes | 30% | 5 |
| Österberg et al. [47] | 2000 | Sweden | 5000 | both | 18 – 45 | Urban | Cross-sectional | Rome | Self-developed, GHQ-20 | No | m: 7.4% (6.0%–8.9%), w: 13.3% (11.5%–5.0%) | 6 |
| Boekema et al. [48] | 2001 | Netherlands | 500 | both | ≥ 18 | Urban | Cross-sectional | Manning | Questionnaire according to Talley [49] | Yes | 5.8% | 7 |
| Mearin et al. [50] | 2001 | Spain | 2000 | both | ≥18 | Diverse | Cross-sectional | Rome ll | Self-developed | Yes | Manning: 10.3%,  Rome I: 12.1 % | 7 |
| Badia et al. [51] | 2002 | Spain | 2000 | both | ≥18 | Diverse | Cross-sectional | Rome l, Rome II | Self-developed, HR-QOL, SF-36 | No | 12.1%, 3.3% | 5 |
| Baretić et al. [52] | 2002 | Croatia | 500 | both |  | Urban | Cross-sectional | Rome |  | No | 28% | 7 |
| Bommelaer et al. [53] | 2002 | France | 11131 | both | ≥18 | Diverse | Cross-sectional | Rome l | Self-developed | No | 4% (95% CI: 3.6%-4.4%) | 7 |
| Icks et al. [54] | 2002 | Germany | 1281 | both | 21 – 80 | Urban | Cross-sectional | Defintion according to Talley, Thompson | Self-developed | No | 12.5 % (10.7-14.5 %) | 6 |
| Hungin et al. [55] | 2003 | UK, France, Germany, Italy, Holland, Belgium, Spain and Switzerland | 41984 | both | ≥18 | Diverse | Cross-sectional | Manning,  Rome l,  Rome ll | Self-developed | Yes | 11.5% (6.2-12%) | 8 |
| Bommelaer et al. [56] | 2004 | France | 8221 | both | ≥18 | Diverse | Cross-sectional | Manning,  Rome l,  Rome ll | Self-developed | Yes | 2.5%,  2.1%,  2.2% | 5 |
| Corazziari et al. [57] | 2004 | Italy | 46139 | both | 30 – 69 | Diverse | Cross-sectional | Rome I | Self-developed | Yes | m: 37.5%.  w: 62.5% | 7 |
| Dapoigny et al. [58] | 2004 | France | 20000 | both | ≥18 | Diverse | Cross-sectional | Rome II | Self-developed questionnaire | Yes | 4.7% (4.36-5.04%) | 9 |
| Hillilä et al. [59] | 2004 | Finnland | 5000 | both | 18–64 | Diverse | Cross-sectional | Manning 2,  Manning 3,  Rome l,  Rome ll | Rome II Integrative Questionnaire, BDI, | No | 16.2%(15.0-17.4),  9.7%(8.8-10.7),  5.5%(4.8-6.3), 5.1%(4.4-5.8) | 8 |
| Vandvik et al. [60] | 2006 | Norway | 4622 | both | 30 – 75 | Urban | Cross-sectional | Rome II | Self-developed, SCL-10 | No | 8.4% (95% CI: 7.6-9.4%) | 7 |
| Aggarwal et al. [21] | 2006 | UK | 2299 | both | 18 – 75 | Urban | Cross-sectional | Rome II | Self-developed, HADS, Health anxiety questionnaire, Sleep problem scale, Somatic symptom checklist | No | 9% | 7 |
| Klooker et al. [61] | 2009 | Netherlands | 1423 | both | 58 | Urban | Longitudinal | Rome II | Rome II questionnaire, HADS, Stress test | No | 9.6 % | 6 |
| Kato et al. [3] | 2009 | Sweden | 31318 twins | both | 41 – 64 | Diverse | Cross-sectional | Swedish Twin Registry criteria (equal to Rome) | Self-developed | Yes | m: 5.1%, w: 8.9%, | 7 |
| Usai et al. [62] | 2010 | Italy | 1900 | both | ≥18 | Diverse | Cross-sectional | Rome ll | Self-developed | Yes | Rural: 9.9%,  Urban,  4.4% | 4 |
| Fosnes et al. [63] | 2011 | Norway | 4622 | both | ≥31 | Diverse | Cross-sectional | Rome II | Self-developed | No | 8.4% | 7 |
| Ziółkowski et al. [64] | 2012 | Poland | 850 | both | 21 – 76 | Urban | Cross-sectional | Manning | Self-developed | Yes | 12.9% | 5 |
| Krogsgaard et al. [65] | 2013 | Denmark | 19657 | both | 18 – 50 | Diverse | Cross-sectional | Rome lll | Self-developed | No | 16% | 7 |
| Kjellström et al. [66] | 2014 | Sweden | 3347 | both | 18 – 70 | Diverse | Cross-sectional | Rome ll | ASQ, Self-developed | Yes | ASQ:  26.2% [95% (CI): 24.4-28.0],  ASQ Coloscopy: 36.6% (95% CI: 33.2-40.1), ASQnon responders: (15.8%; 95% CI: 11.4-20.3)  Rome ll: 14.8% (95% CI: 12.2-17.5), Rome ll coloscopy:  14.5% (95% CI: 11.9-17.2) | 8 |
| Janssens et al. [5] | 2015 | Netherlands | 94516 | both | 25-50 | Diverse | Cross-sectional | DSM-IV and ICD-10 | MINI | yes | 9.7% | 8 |
| Krogsgaard et al. [67] | 2017 | Denmark | 19657 | both | 18 – 50 | Diverse | Cross-sectional | Rome lll | Self-developed | No | 2010: 17.0%(15.9-18.3), 2011:  19.6%(18.2-21.0) | 8 |
| Van den Houte et al. [68] | 2019 | Belgium | 1012 | both | ≥18 | Diverse | Cross-sectional | Rome IV | Self-developed | No | 5.5% | 5 |
| Schauer et al. [69] | 2019 | Germany | 4194 | both | 20 – 79 | Urban | Longitudinal | Rome lll | Self-developed, PHQ-9, BDI-2, TAS-20, CTQ, SF-12, FFS | No | 3.5% (3.0%-4.2%) | 7 |
| Nakov et al. [70] | 2020 | Bulgaria | 1896 | both | 18 – 65 | Diverse | Cross-sectional | Rome Vl | Self-developed | No | 20% | 4 |
| Petersen et al. [6] | 2020 | Denmark | 9656 | both | 18 – 76 | Diverse | Cross-sectional | According to diagnostic system | Self-developed, SF-36 | No | 3.6% (3.2-3.9) | 7 |
| Petersen et al. [7] | 2020 | Denmark | 1590 | both | 44 – 63 | Diverse | Cross-sectional | EURO-SOMA | Self-developed | Yes | 3.8% (3.1–4.6) | 9 |
| Sperber et al. [71] | 2021 | Belgium,  France,  Germany,  Netherlands,  Italy,  Sweden,  Spain,  Poland,  UK | 2021,  2019,  2020,  2008,  2063,  2084,  2072,  2057,  2027 | both | ≥18 | Diverse | Cross-sectional | Rome IV | Self-developed | No | 3.3% (2.5–4.0),  4.2 (3.3–5.0),  3.7 (2.8–4.5),  3.8 (2.9–4.6),  5.0 (4.1–5.9),  4.0 (3.1–4.8),  4.2 (3.4–5.1),  4.0 (3.1–4.8)  4.4 (3.5–5.3) | 8 |
| Results for chronic pain | | | | | | | | | | | | |
| Brattberg et al. [72] | 1989 | Sweden | 1009 | both | 18 – 84 | Urban | Cross-sectional | IASP | Self-developed | No | 40% | 6 |
| Chrubasik et al. [73] | 1998 | Germany | 1304 | both | 18 – 80 | Diverse | Cross-sectional | Definition: 6 months | Self-developed | No | 14.3%  (12.4-16.2) | 6 |
| Catala et al. [74] | 2002 | Spain | 1546 | both | 18 – 95 | Diverse | Cross-sectional | IASP | Self-developed | Yes | 23.4% | 6 |
| Rustøen et al. [75] | 2004 | Norway | 4000 | both | 19 – 81 | Diverse | Cross-sectional | IASP | Self-developed, Brief Pain Inventory | No | 24.4% | 7 |
| Breivik et al. [76] | 2006 | Spain,  Ireland,  UK,  France,  Switzerland,  Denmark,  Germany,  Netherlands,  Sweden,  Finland, Austria, Belgium, Italy,  Poland, Norway | Spain: 3801,  Ireland: 2722,  UK: 3800,  France: 3846,  Switzerland: 2083,  Denmark: 2169,  Germany: 3832,  Netherlands: 3197,  Sweden: 2563,  Finland: 2004, Austria: 2004, Belgium: 2451, Italy: 3849,  Poland: 3812, Norway: 2018 | both | ≥18 | Diverse | Cross-sectional | Definition: pain duration more than 6 months | CATI | Yes | Spain: 12.0%,  Ireland 13.0%,  UK: 13.0%,  France 15.0%,  Switzerland 16.0%,  Denmark 16.0%,  Germany 17.0%,  Netherlands 18.0%,  Sweden 18.0%,  Finland 19.0%, Austria 21.0%, Belgium 23.0%, Italy 26.0%,  Poland 27.0%, Norway 30.0% | 5 |
| Jablonska et al. [77] | 2006 | Sweden | 3616 | only females | 18 – 64 | Urban | Cross-sectional | Definition: 3 months | The Pain Questionnaire | No | 40.1% | 6 |
| Bouhassira et al. [78] | 2008 | France | 23712 | both | ≥18 | Diverse | Cross-sectional | Definition: 3 months | Brief Pain Inventory, NRS | No | 31.7%; (95%CI: 31.1–32.3) | 8 |
| Landmark et al. [79] | 2012 | Norway | 94194 | both | ≥20 | Urban | Longitudinal | Definition: 6 months | Self-developed, SF-8 | No | Definition by duration: 47% (95% CI 45 to 49),  SF-8 : 33% (95% CI 32 to 35), SF8 and Duration:  28% (95% CI 27 to 30) | 8 |
| Azevedo et al. [80] | 2012 | Portugal | 5094 | both | ≥18 | Urban | Cross-sectional | IASP | Self-developed | Yes | 36.7% (95%[35.3-38.2] | 9 |
| Björnsdóttir et al. [81] | 2013 | Iceland | 5906 | both | 18 – 79 | Diverse | Cross-sectional | IASP | Self-developed, SF-36,  IPAQ, | No | 19.9% | 8 |
| Dueñas et al. [82] | 2015 | Spain | 1957 | both | ≥18 | Diverse | Cross-sectional | IASP | Self-developed | Yes | 16.6% (95%: 14.9–18.3) | 8 |
| Del Giorno et al. [83] | 2017 | Italy | 1293 | both | ≥18 | Diverse | Cross-sectional | Definition: 3 months | Self-developed | No | 28.4% | 5 |
| Musculoskeletal pain | | | | | | | | | | | | |
| Bergman et al. [84] | 2001 | Sweden | 70704 | both | 20 – 74 | Urban | Cross-sectional | ACR 1990 criteria | Self-developed, SF-36 | No | 23.9% | 8 |
| Wijnhoven et al. [85] | 2006 | Netherlands | 7836 | both | 25- 64 | Diverse | Cross-sectional | Definition: 3 months | Self-developed | Yes | M39%, W 45%, | 7 |
| Hagen et al. [86] | 2011 | Norway | 92936,  94194 | both | ≥20 | Urban | Longitudinal cohort | ACR 1990 criteria | Self-developed, HADS | Yes | 44.8%, 95% CI 44.5-45.2,  47.9%, 95% CI 47.6-48.2 | 9 |
| MacFarlane et al. [87] | 2015 | UK | 503325 | both | 40 – 69 | Diverse | Cross-sectional | Definition: 3 months | Self-developed | No | 42.9%, 99% CI: 42.7%, 43.1% | 6 |
| Pelvic pain | | | | | | | | | | | | |
| Zondervan et al. [88] | 2001 | UK | 3916 | females | 18 – 49 | Diverse | Cross-sectional | Definition: 3 months | Self-developed | No | 24% | 6 |
| Mohedo et al. [89] | 2014 | Spain | 940 | females | 18 – 65 | Urban | Cross-sectional | Definition: 6 months | Self-developed: CPPQ-Mohedo | No | 26.8% | 7 |
| Margueritte et al. [90] | 2021 | France | 24763 | females | 18 – 49 | Diverse | Cross-sectional | Open question | Self-developed | No | 17.0% (95% CI, 16.5–17.5) | 7 |
| Abdominal pain: | | | | | | | | | | | | |
| Icks et al. [54] | 2002 | Germany | 1281 | both | 21 – 80 | Urban | Cross-sectional | Defintion according to Talley, Thompson | Self-developed | No | 22.6 % (95 %-CI: 20.3 - 25.1 %) | 6 |
| LBP: | | | | | | | | | | | | |
| Hillman et al. [91] | 1996 | UK | 3184 | both | 25 – 64 | Urban | Cross-sectional | Definition: 12 months | Self-developed | No | 19% | 6 |
| Smith et al. [92] | 2004 | Scotland | 2184 | both | ≥25 | Diverse | Longitudinal | Definition: 3 months | CPG,  LEN, SF-36 | No | 1996:  16%,  2000: 27% | 7 |
| Bjorck-Van Dijken et al. [93] | 2008 | Sweden | 5798 | both | 25 – 79 | Diverse | Cross-sectional | Definition: 6 months | (WHO) MONICA | No | 41% | 7 |
| Heuch et al. [94] | 2010 | Norway | 63968 | both | ≥20 | Diverse | Cross-sectional | Definition: 3 months | Self-developed | No | m: 20.9%,  w: 26.3% | 6 |
| Gerhardt et al. [26] | 2014 | Germany | 4000 | both | ≥18 | Diverse | Longitudinal | Definition: 3 months,  DSM-IV | Self-developed, SF-36. PHQ-D | No | 17.7% | 7 |
| Gouveia et al. [95] | 2016 | Portugal | 10661 | both | ≥18 | Urban | Cross-sectional | Definition: 90 days | Self-developed | Yes | 10.4% (9.6; 11.9) | 9 |
| Ho et al. [96] | 2019 | Netherlands | 50666 | both | 20 – 96 | Rural | Cross-sectional | Definition: 3 months | Self-developed | No | 21.4% | 7 |
| Miscellaneous pain: | | | | | | | | | | | | |
| Mäkela et al. [97]  *Chronic neck pain* | 1991 | Finnland | 8000 | both | ≥30 | Diverse | Cross-sectional | Definition: 3 month | Mini-Finland Health Survey | Yes | m: 9.5%,  w: 13.5% | 8 |
| Guez et al. [98]  *Chronic neck pain* | 2002 | Sweden | 6000 | both | 25 – 74 | Urban | Cross-sectional | Definition: 6 months | (WHO) MONICA | No | m: 16%,w: 22% | 7 |
| Guez et al. [99]  *Chronic neck pain* | 2003 | Sweden | 4415 | both | 25 – 64 | Urban | Cross-sectional | Definition: 6 months | (WHO) MONICA | Yes | 18% | 8 |
| Leijon et al. [100]  *Neck shoulder arm pain* | 2009 | Sweden | 1990: 1976,  1994: 8424,  1998: 2435,  2002: 24430,  2006: 26611 | both | 21– 64 | Diverse | Cross-sectional | Definition: 6 (12) months | Stockholm Public Health (SPH) Questionnaire | No | 1990: w: 22.8 (20.2;25.3); m: 12.8 (10.6;14.9)  1994: w: 22.9 (21.6;24.1); m: 14.1 (13.0;15.2),  1998: w: 26.0 (23.7;28.4); m: 16.6 (14.5;18.8),  2002: w: 30.7 (29.9;31.5); m: 19.1 (18.3;19.8),  2006: w: 24.9(24.2;25.6); m: 14.9 (14.2;15.5) | 8 |
| Wertli et al. [101]  *Non-cardiac chest pain* | 2019 | Switzerland | 1341 | both | ≥18 | Urban | Cross-sectional | ICD-10 | ICD-10 codes analyzes | No | 44.7% | 5 |
| Results for miscellaneous functional disorders | | | | | | | | | | | | |
| Ribas et al. [102]  *Functional constipation* | 2011 | Spain | 1020 | only females | 18 – 45 | Urban | Longitudinal cohort | Rome II | Self-developed, Patient records | No | 28.8% | 5 |
| Fosnes et al. [63]  *Functional constipation* | 2011 | Norway | 4622 | both | ≥31 | Diverse | Cross-sectional | Rome II | Self-developed | No | 11.6% | 7 |
| Walsh et al. [103]  *Functional constipation* | 2012 | Ireland | 1909 | both | 20 – 89 | Diverse | Cross-sectional | ICD-10, Rome III | Patients records | No | 22.5% | 7 |
| Rey et al. [104]  *Chronic constipation* | 2014 | Spain | 1500 | both | ≥18 | Diverse | Cross-sectional | Rome III | Rome III questionnaire | No | 19.2% (17.2 – 21.2) | 8 |
| Enck et al. [105]  *Functional constipation* | 2016 | Germany | 15000 | both | ≥18 | Diverse | Cross-sectional | Rome III | Rome III questionnaire | No | 6.2% | 6 |
| Pannemans et al. [106]  *Chronic constipation* | 2020 | Belgium | 1012 | both | ≥18 | Diverse | Cross-sectional | Rome IV | Self-developed | No | 21% | 5 |
| Breckan et al. [107]  *Functional bowel symptoms* | 2012 | Norway | 1416 | both | 18 – 85 | Urban | Cross-sectional | Rome II | GSRS | No | 25.1% | 7 |
| Dantoft et al. [108]  *Multiple chemical sensitivity* | 2021 | Denmark | 9656 | both | 18 – 76 | Diverse | Cross-sectional | 1999 US Consensus Criteria for MCS | Self-developed | Yes | 1.95% | 8 |
| Petersen et al. [6]  *Multiple chemical sensitivity* | 2020 | Denmark | 9656 | both | 18 – 76 | Diverse | Cross-sectional | According to diagnostic system | Self-developed, SF-36 | No | 2.0% (1.7-2.3) | 7 |
| Petersen et al. [7]  *Multiple chemical sensitivity* | 2020 | Denmark | 1590 | both | 44 – 63 | Diverse | Cross-sectional | EURO-SOMA | Self-developed | Yes | 0.9% (0.6–1.3) | 9 |
| Wenzel et al. [109]  *Whiplash associated disorders* | 2009 | Norway | 55046 | both | ≥20 | Diverse | Cross-sectional | Self-developed: whiplash and 12 months pain duration | Self-developed | No | Total number: 785,  1.43% | 7 |
| Petersen et al. [6]  *Whiplash associated disorders* | 2020 | Denmark | 9656 | both | 18 – 76 | Diverse | Cross-sectional | According to diagnostic system | Self-developed, SF-36 | No | 1.7% (1.4-1.9) | 7 |
| Petersen et al. [7]  *Whiplash associated disorder* | 2020 | Denmark | 1590 | both | 44 – 63 | Diverse | Cross-sectional | EURO-SOMA | Self-developed | Yes | 1.5% (1.0–2.0) | 9 |
| Olafsdottir et al. [110]  *Functional dyspepsia* | 2010 | Iceland | 2000 | both | 18 – 75 | Diverse | Longitudinal cohort | Rome III | Functional Dyspepsia Score List | No | 1996: 13.9%,  2006: 16.7% | 7 |
| Nakov et al. [70]  *Functional dyspepsia* | 2020 | Bulgaria | 1896 | both | 18 – 65 | Diverse | Cross-sectional | Rome IV and criteria depending on duration and symptoms | Self-developed | No | 12.7% | 4 |
| Zagari et al. [111]  *Functional dyspepsia* | 2010 | Italy | 1033 | both | 18 – 69 | Urban | Cross-sectional | Modified Rome II | Self-developed | Yes | 11% (95% CI, 9.2–12.9) | 9 |
| Walsh et al. [103]  *Functional dyspepsia* | 2012 | Ireland | 1909 | both | 20 – 89 | Diverse | Cross-sectional | ICD-10, Rome III | Patients records | No | 21.0% | 7 |
| Ebling et al. [112]  *Functional dyspepsia* | 2016 | Croatia | 9002 | both | 20 – 69 | Urban | Cross-sectional | Rome III | Rome III diagnostic questionnaire | No | 16.56% | 7 |
| Walsh et al. [103]  *Functional heartburn* | 2012 | Ireland | 1909 | both | 20 – 89 | Diverse | Cross-sectional | ICD-10, Rome III | Patients records | No | 5.6% | 7 |
| Walsh et al. [103]  *Functional vomiting* | 2012 | Ireland | 1909 | both | 20 – 89 | Diverse | Cross-sectional | ICD-10, Rome III | Patients records | No | 1.9% | 7 |
| Walsh et al. [103]  *Functional dysphagia* | 2012 | Ireland | 1909 | both | 20 – 89 | Diverse | Cross-sectional | ICD-10, Rome III | Patients records | No | 1.5% | 7 |
| Fosnes et al. [63]  *Functional diarrhoea* | 2011 | Norway | 4622 | both | ≥31 | Diverse | Cross-sectional | Rome II | Self-developed | No | 6.4% | 7 |
| Walsh et al. [103]  *Functional diarrhea* | 2012 | Ireland | 1909 | both | 20 – 89 | Diverse | Cross-sectional | ICD-10, Rome III | Patients records | No | 1.1% | 7 |
| Sperber et al. [71]  *Functional Diarrhea* | 2021 | Belgium,  France,  Germany,  Netherlands,  Italy,  Sweden,  Spain,  Poland,  UK | 2021,  2019,  2020,  2008,  2063,  2084,  2072,  2057,  2027 | both | ≥18 | Diverse | Cross-sectional | Rome IV | Self-developed | No | 4.0 (3.2–4.9),  6.1 (5.1–7.2),  5.4 (4.4–6.4),  3.2 (2.5–4.0),  3.2 (2.5–4.0),  5.9 (4.8–6.9),  4.8 (3.9–5.7),  4.5 (3.6–5.4),  4.5 (3.6–5.4) | 8 |
| Walsh et al. [103]  *Functional galbladder/sphincter of oddi dysfunction* | 2012 | Ireland | 1909 | both | 20 – 89 | Diverse | Cross-sectional | ICD-10, Rome III | Patients records | No | 0.7% | 7 |
| Walsh et al. [103]  *Functional incontinence* | 2012 | Ireland | 1909 | both | 20 – 89 | Diverse | Cross-sectional | ICD-10, Rome III | Patients records | No | 0.7% | 7 |
| Maaranen et al. [113]  *Dissociation* | 2008 | Finnland | 1497 | both | 26 – 65 | Urban | Cross-sectional | DSM-IV | DES | No | 2.0%, | 7 |
| Sperber et al. [71]  *Functional dyspepsia* | 2021 | Belgium,  France,  Germany,  Netherlands,  Italy,  Sweden,  Spain,  Poland,  UK | 2021,  2019,  2020,  2008,  2063,  2084,  2072,  2057,  2027 | both | ≥18 | Diverse | Cross-sectional | Rome IV | Self-developed | No | 5.0 (4.0–5.9),  8.5 (7.3–9.7),  6.9 (5.8–8.0),  4.1 (3.2–5.0),  9.1 (7.8–10.3),  8.2 (7.0–9.4),  7.4 (6.3–8.5), 8.3 (7.1–9.5), 6.6 (5.5–7.6) | 8 |
| Sperber et al. [71]  *Functional constipation* | 2021 | Belgium,  France,  Germany,  Netherlands,  Italy,  Sweden,  Spain,  Poland,  UK | 2021,  2019,  2020,  2008,  2063,  2084,  2072,  2057,  2027 | both | ≥18 | Diverse | Cross-sectional | Rome IV | Self-developed | No | 11.0 (9.7–12.4),  14.5 (12.6–16.1),  9.8 (7.9–10.5),  9.2 (7.9–10.5),  14.4 (12.7–15.8),  10.3 (9.0–11.6),  12.8 (11.4–14.3),  14.2 (12.7–15.8),  8.6 (7.4–9.8) | 8 |
| Sperber et al. [71]  *Functional Bloating, distension* | 2021 | Belgium,  France,  Germany,  Netherlands,  Italy,  Sweden,  Spain,  Poland,  UK | 2021,  2019,  2020,  2008,  2063,  2084,  2072,  2057,  2027 | both | ≥18 | Diverse | Cross-sectional | Rome IV | Self-developed | No | 2.4 (1.7–3.0),  6.0 (5.0–7.0),  2.8 (2.1–3.5),  1.5 (1.0–2.0),  8.2 (7.1–9.4),  3.1 (2.4–3.9), 3.4 (2.6–4.2),  5.3 (4.3–6.3),  3.8 (3.0–4.7) | 8 |

*Note.* DSM-IV = Diagnostic and Statistical Manual of Mental Disorders, 4^th^ revision, ICPC = International Classification of Primary Care, SF-12 = Short Form 12 item questionnaire, HAQ = Health Assessment Questionnaire, BL scale = List of complaints (von Zerssen D, Koeller D-M. Die Beschwerden-Liste); FFbH-R = Der Funktionsfragebogen Hannover zur alltagsnahen Diagnostik der Funktionsbeeinträchtigung durch Rückenschmerzen Hannover; Functional Questionnaire in ambulatory diagnosis of functional disability caused by backache. Rehabilitation 1996;35:I–VIII, FSQ = Fibromyalgia Survey Questionnaire, MINI = Mini International Neuropsychiatric Interview 5.0.0, CFQ = Chalder Fatigue Questionnaire, GHQ = General Health Questionnaire (Goldberg & Williams, 1988), GSRS = Gastrointestinal symptom rating scale Present State Examination and Psychiatric Symptom Frequency scale, SFQ = Shortened fatigue questionnaire, CIS-R = Revised Clinical Interview Schedule, MOS = Medical Outcomes Study Health Survey Short Form, ASQ = Abdominal Symptom Questionnaire, HSCL-25 = 25-item Hopkins Symptom Checklist, CIDI = Composite International Diagnostic Interview, DIA-X/M-CIDI = Munich-Composite International Diagnostic Interview, CSR-S = Clinical Self Rating Scales, GHQ-20 = Goldberg & Hillier, 1979; Lobo et al, 1986, CAGE questionnaire, MMSE = Mini Mental State Examination, SPPI = Standardised Polyvalent Psychiatric Interview, ICPC-2-E = International Classification of Primary Care, DDPRQ-10 = Difficult Doctor-Patient Relationship Questionnaire, SPPI = Standardized Polyvalent Psychiatric Interview, CSRI = Client Service Receipt Inventory, BDQ = Bowel Disease Questionnaire, EQ-5D = EuroQol with five domains measure of health status, LFESSQ = London Fibromyalgia Epidemiology Symptom Screening questionnaire, CPPQ-M = Chronic Pelvic Pain Questionnaire – Mohedo, IPAQ = International Physical Activity Questionnaire, CPG = Chronic Pain Grade questionnaire, LEN = Level of Expressed Needs, CDQ = case definition questionnaire, PQ = The Pain Questionnaire, ICPC = International Classification of Primary Care, DES = Dissociative Experiences Scale, DES-T = DES-T DES-taxon (ergänzen), BPI = Brief Pain Inventory, BQ = Berlin Questionnaire

# Table S2. PubMed search strategy with search terms applied with filters: adults > 19 years, European countries

| Group 1 | (((((((“Functional disorder”) OR ("functional somatic*")) OR ("functional symptom*")) OR ("functional syndrome*")) OR ("functional symptomatic")) OR ("functional illness*")) OR (Functional limitation*)) OR ("functional disease*") |
| --- | --- |
| Group 2 | ((((((((((((bodily distress) OR (somatoform)) OR (dissociat*)) OR (conversion)) OR (Somatic symptom)) OR (Illness anxiety)) OR (Hypochond*)) OR (somatizer*)) OR (hysteri*)) OR ("somatic symptom and related")) OR (SSD)) OR (Neurasthenia)) OR (briquet) |
| Group 3 | ((((((((((((((((((((Medically unexplained) OR ("organically unexplain*")) OR (psychophysiological disorder)) OR (psychosomatic medicine)) OR (psychosomat*)) OR (psychogen*)) OR ("persistent physical symptom*")) OR ("persistent somatic symptom")) OR ("physical symptom disorder")) OR (multisomat*)) OR (polysymptom*)) OR (MUS)) OR (MUPS)) OR (FSS)) OR ("stress disorder*")) OR ("distress disorder*")) OR ("psychological factors affecting med*")) OR ("unexplained medical*")) OR ("unexplained symptom*")) OR ("multiple physical symptom*")) OR ("multiple symptom* diagnos*") |
| Group 4 | ((((((((((((Irritable bowel syndrome*) OR (“functional gastro-intestinal")) OR ("functional intestinal")) OR (“functional esophageal")) OR ("functional constipation")) OR ("functional dyspepsia")) OR ("functional diarrhea")) OR ("functional urinary")) OR ("functional colonic disease*")) OR (IBS)) OR (heart neurosis)) OR (“functional cardiovascular")) OR ("functional palpitation") |
| Group 5 | ((((((((((((((((persistent pain) OR (chronic pain)) OR (Fibromy*)) OR (tension headache*)) OR ("chronic musculoskeletal pain")) OR ("functional abdominal pain")) OR ("tension type headache")) OR (fibrositis)) OR (fibromyositis)) OR (myofibrositis)) OR ("chronic widespread pain")) OR ("widespread musculoskeletal pain")) OR ("myofascial pain")) OR ("chronic intractable benign pain*")) OR (non cardiac chest pain)) OR (non specific chest pain)) OR (atypical pain) |
| Group 6 | (((((((((((((((((Sensory disorder) OR (functional neurological)) OR ("functional movement")) OR ("functional epileptic seizures")) OR ("dissociative seizures")) OR ("psychogenic epileptic seizure*")) OR (Functional cognitive disorder*)) OR ("functional dizziness")) OR ("Functional Paresthesia")) OR ("psychogenic pruritus")) OR (non-epileptic seizures)) OR (chronic fatigue syndrome*)) OR ("Myalgic encephalomyelitis")) OR ("psychogenic fatigue")) OR ("myalgic encephalomyelitis*")) OR ("myalgic encephalopath*")) OR (CFS)) OR ("CFS/ME") |
| Group 7 | (Prevalence*) OR (Epidemiol*) |
| Results: | #1 OR #2 OR #3 OR #4 OR #5 AND #7 |

# Figures

>>>>>>>>>>>>>>>>insert S1 here <<<<<<<<<<<<<<<<<<<<<<<<<<

Figure S1. Forest plot of overall point prevalence 95% Confidence interval and prediction interval. Studies on all functional disorders are represented

*Note.* AT = Austria, BE = Belgium, BG = Bulgaria, CH = Switzerland, DE = Germany, DK = Denmark, ES = Spain, FI = Finland, FR = France, GB = Great Britain, HR = Croatia, IE = Ireland, IT = Italy, IS = Island, NL = The Netherlands, NO = Norway, PL = Poland, PT = Portugal, SE = Sweden, AP = Abdominal pain, CNP = Chronic neck pain, CP = Chronic pain, CFS = Chronic fatigue syndrome, CHP = Chest pain, CWP = Chronic widespread pain, FBLO = Functional bloating, FBOW = Functional bowel symptoms, FCON = Functional constipation, FDIAR = Functional diarrhea, FDYS = Functional dyspepsia, FDPH = Functional dysphagia, FGAL = Functional gallbladder, FINCON= Functional incontinence, FVOM = Functional vomiting, FM = Fibromyalgia, IBS = Irritable Bowel Syndrome, LBP = Low back pain, MCS= Multiple chemical sensitivity, PAIN = musculoskeletal pain, PP = Pelvic pain, SOM = Somatization, TTH = Tension type headaches, WAD = Whiplash associated disorder


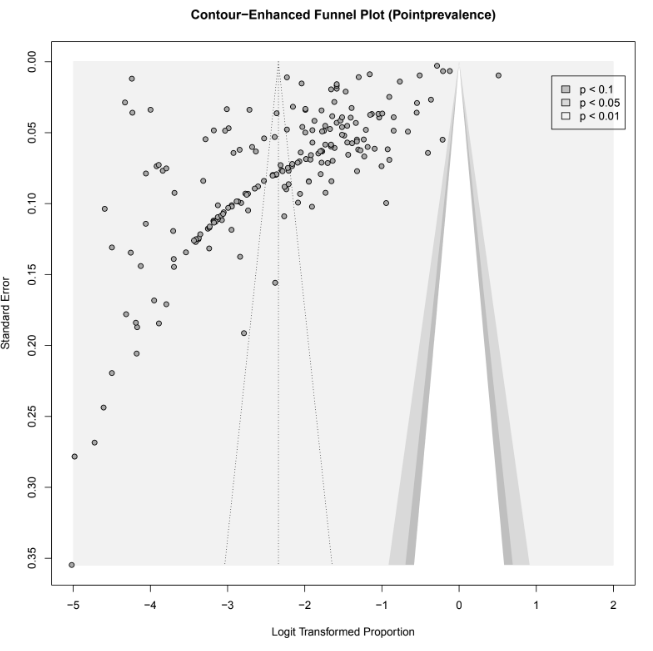


Figure S2. Funnel Plot of point prevalence studies of functional disorders, findings show a significant asymmetry

>>>>> insert Fig. S3 here <<<<

Figure S3. Forest plot of specific diagnosis on country level with 95% confidence intervals and prediction intervals in regard to the author, year of publication, and country

*Note.* CP = Chronic pain, CFS = Chronic fatigue syndrome, CWP = Chronic widespread pain, FM = Fibromyalgia, IBS = Irritable Bowel Syndrome, SOM = Somatization,

# References:

1. Líndal E, Stefánsson JG, Bergmann S: The prevalence of chronic fatigue syndrome in Iceland-a national comparison by gender drawing on four different criteria. *Nordic journal of psychiatry* 2002, 56:273-277.

2. Harvey SB, Wadsworth M, Wessely S, Hotopf M: Etiology of chronic fatigue syndrome: testing popular hypotheses using a national birth cohort study. *Psychosomatic Medicine* 2008, 70:488-495.

3. Kato K, Sullivan PF, Evengård B, Pedersen NL: A population-based twin study of functional somatic syndromes. *Psychological medicine* 2009, 39:497-505.

4. van’t Leven M, Zielhuis GA, van der Meer JW, Verbeek AL, Bleijenberg G: Fatigue and chronic fatigue syndrome-like complaints in the general population. *European journal of public health* 2010, 20:251-257.

5. Janssens KA, Zijlema WL, Joustra ML, Rosmalen JG: Mood and anxiety disorders in chronic fatigue syndrome, fibromyalgia, and irritable bowel syndrome: results from the LifeLines cohort study. *Psychosomatic medicine* 2015, 77:449-457.

6. Petersen MW, Schröder A, Jørgensen T, Ørnbøl E, Dantoft TM, Eliasen M, Carstensen TW, Falgaard Eplov L, Fink P: Prevalence of functional somatic syndromes and bodily distress syndrome in the Danish population: the DanFunD study. *Scandinavian journal of public health* 2020, 48:567-576.

7. Petersen MW, Schröder A, Jørgensen T, Ørnbøl E, Meinertz Dantoft T, Eliasen M, Benros ME, Fink P: Irritable bowel, chronic widespread pain, chronic fatigue and related syndromes are prevalent and highly overlapping in the general population: DanFunD. *Scientific reports* 2020, 10:1-10.

8. Mäkelä M, Heliövaara M: Prevalence of primary fibromyalgia in the Finnish population. *British Medical Journal* 1991, 303:216-219.

9. Forseth K, Gran J: The prevalence of fibromyalgia among women aged 20–49 years in Arendal, Noway. *Scandinavian journal of rheumatology* 1992, 21:74-78.

10. Prescott E, Kjøller M, Jacobsen S, Bülow P, Danneskiold-Samsøe B, Kamper-Jørgensen F: Fibromyalgia in the adult Danish population: I. A prevalence study. *Scandinavian journal of rheumatology* 1993, 22:233-237.

11. Lindell L, Bergman S, Petersson IF, Jacobsson LT, Herrström P: Prevalence of fibromyalgia and chronic widespread pain. *Scandinavian journal of primary health care* 2000, 18:149-153.

12. Carmona L, Ballina J, Gabriel R, Laffon A: The burden of musculoskeletal diseases in the general population of Spain: results from a national survey. *Annals of the rheumatic diseases* 2001, 60:1040-1045.

13. Schochat T, Raspe H: Elements of fibromyalgia in an open population. *Rheumatology* 2003, 42:829-835.

14. Mas A, Carmona L, Valverde M, Ribas B: Prevalence and impact of fibromyalgia on function and quality of life in individuals from the general population: results from a nationwide study in Spain. *Clinical & Experimental Rheumatology* 2008, 26:519.

15. Perrot S, Vicaut E, Servant D, Ravaud P: Prevalence of fibromyalgia in France: a multi-step study research combining national screening and clinical confirmation: The DEFI study (Determination of Epidemiology of FIbromyalgia). *BMC musculoskeletal disorders* 2011, 12:1-9.

16. Lourenço S, Costa L, Rodrigues AM, Carnide F, Lucas R: Gender and psychosocial context as determinants of fibromyalgia symptoms (fibromyalgia research criteria) in young adults from the general population. *Rheumatology* 2015, 54:1806-1815.

17. Jones GT, Atzeni F, Beasley M, Flüß E, Sarzi‐Puttini P, Macfarlane GJ: The prevalence of fibromyalgia in the general population: a comparison of the American College of Rheumatology 1990, 2010, and modified 2010 classification criteria. *Arthritis & rheumatology* 2015, 67:568-575.

18. Gayà TF, Ferrer CB, Mas AJ, Seoane-Mato D, Reyes FÁ, Sánchez MD, Dubois CM, Sánchez-Fernández SA, Vargas LMR, Morales PG: Prevalence of fibromyalgia and associated factors in Spain. *Clin Exp Rheumatol* 2020, 123:47-52.

19. Croft P, Rigby A, Boswell R, Schollum J, Silman A: The prevalence of chronic widespread pain in the general population. *The Journal of rheumatology* 1993, 20:710-713.

20. Bergman S, Herrström P, Jacobsson LT, Petersson IF: Chronic widespread pain: a three year followup of pain distribution and risk factors. *The Journal of rheumatology* 2002, 29:818-825.

21. Aggarwal VR, McBeth J, Zakrzewska JM, Lunt M, Macfarlane GJ: The epidemiology of chronic syndromes that are frequently unexplained: do they have common associated factors? *International journal of epidemiology* 2006, 35:468-476.

22. Gerdle B, Björk J, Cöster L, Henriksson K-G, Henriksson C, Bengtsson A: Prevalence of widespread pain and associations with work status: a population study. *BMC musculoskeletal disorders* 2008, 9:1-10.

23. VanDenKerkhof EG, Macdonald HM, Jones GT, Power C, Macfarlane GJ: Diet, lifestyle and chronic widespread pain: results from the 1958 British Birth Cohort Study. *Pain Research and Management* 2011, 16:87-92.

24. Gale CR, Deary IJ, Cooper C, Batty GD: Intelligence in childhood and chronic widespread pain in middle age: the National Child Development Survey. *PAIN®* 2012, 153:2339-2344.

25. Creed F, Tomenson B, Chew-Graham C, Macfarlane G, Davies I, Jackson J, Littlewood A, McBeth J: Multiple somatic symptoms predict impaired health status in functional somatic syndromes. *International journal of behavioral medicine* 2013, 20:194-205.

26. Gerhardt A, Hartmann M, Blumenstiel K, Tesarz J, Eich W: The prevalence rate and the role of the spatial extent of pain in nonspecific chronic back pain—a population-based study in the south-west of Germany. *Pain Medicine* 2014, 15:1200-1210.

27. Mundal I, Gråwe RW, Bjørngaard JH, Linaker OM, Fors EA: Prevalence and long-term predictors of persistent chronic widespread pain in the general population in an 11-year prospective study: the HUNT study. *BMC musculoskeletal disorders* 2014, 15:1-12.

28. Burri A, Ogata S, Vehof J, Williams F: Chronic widespread pain: clinical comorbidities and psychological correlates. *Pain* 2015, 156:1458-1464.

29. Flüß E, Bond CM, Jones GT, Macfarlane GJ: The re-evaluation of the measurement of pain in population-based epidemiological studies: The SHAMA study. *British journal of pain* 2015, 9:134-141.

30. Walker-Bone K, Harvey NC, Ntani G, Tinati T, Jones GT, Smith BH, Macfarlane GJ, Cooper C: Chronic widespread bodily pain is increased among individuals with history of fracture: findings from UK Biobank. *Archives of osteoporosis* 2016, 11:1-10.

31. Göbel H, Petersen‐Braun M, Soyka D: The epidemiology of headache in Germany: a nationwide survey of a representative sample on the basis of the headache classification of the International Headache Society. *Cephalalgia* 1994, 14:97-106.

32. Ottar Sjaastad M, Bakketeig LS: Tension-type headache. Comparison with migraine without aura and cervicogenic headache. The Vågå study of headache epidemiology. *Functional neurology* 2008, 23:71.

33. Kristiansen HA, Kværner KJ, Akre H, Øverland B, Russell MB: Tension-type headache and sleep apnea in the general population. *The journal of headache and pain* 2011, 12:63-69.

34. Garcia-Campayo J, Lobo A, Perez-Echeverria MJ, Campos R: Three forms of somatization presenting in primary care settings in Spain. *The Journal of nervous and mental disease* 1998, 186:554-560.

35. Grabe HJ, Meyer C, Hapke U, Rumpf H-J, Freyberger HJ, Dilling H, John U: Specific somatoform disorder in the general population. *Psychosomatics* 2003, 44:304-311.

36. De Waal MWM, Arnold IA, Eekhof JA, Van Hemert AM: Somatoform disorders in general practice: prevalence, functional impairment and comorbidity with anxiety and depressive disorders. *The British Journal of Psychiatry* 2004, 184:470-476.

37. Norton J, De Roquefeuil G, Boulenger J-P, Ritchie K, Mann A, Tylee A: Use of the PRIME-MD Patient Health Questionnaire for estimating the prevalence of psychiatric disorders in French primary care: comparison with family practitioner estimates and relationship to psychotropic medication use. *General hospital psychiatry* 2007, 29:285-293.

38. Hanel G, Henningsen P, Herzog W, Sauer N, Schaefert R, Szecsenyi J, Löwe B: Depression, anxiety, and somatoform disorders: vague or distinct categories in primary care? Results from a large cross-sectional study. *Journal of psychosomatic research* 2009, 67:189-197.

39. Roca M, Gili M, Garcia-Garcia M, Salva J, Vives M, Campayo JG, Comas A: Prevalence and comorbidity of common mental disorders in primary care. *Journal of affective disorders* 2009, 119:52-58.

40. Schaefert R, Laux G, Kaufmann C, Schellberg D, Bölter R, Szecsenyi J, Sauer N, Herzog W, Kuehlein T: Diagnosing somatisation disorder (P75) in routine general practice using the International Classification of Primary Care. *Journal of psychosomatic research* 2010, 69:267-277.

41. Haftgoli N, Favrat B, Verdon F, Vaucher P, Bischoff T, Burnand B, Herzig L: Patients presenting with somatic complaints in general practice: depression, anxiety and somatoform disorders are frequent and associated with psychosocial stressors. *BMC family practice* 2010, 11:1-8.

42. Gaburri M, Bassotti G, Bacci G, Cinti A, Bosso R, Ceccarelli P, Paolocci N, Pelli M, Morelli A: Functional gut disorders and health care seeking behavior in an Italian non-patient population. *Recenti progressi in medicina* 1989, 80:241-244.

43. Heaton KW, O'Donnell LJ, Braddon FE, Mountford RA, Hughes AO, Cripps PJ: Symptoms of irritable bowel syndrome in a British urban community: consulters and nonconsulters. *Gastroenterology* 1992, 102:1962-1967.

44. Agréus L, Svärdsudd K, Nyrén O, Tibblin G: Irritable bowel syndrome and dyspepsia in the general population: overlap and lack of stability over time. *Gastroenterology* 1995, 109:671-680.

45. Agréus L, Talley N, Svärdsudd K, Tibblin G, Jones M: Identifying dyspepsia and irritable bowel syndrome: the value of pain or discomfort, and bowel habit descriptors. *Scandinavian journal of gastroenterology* 2000, 35:142-151.

46. Thompson W, Heaton K, Smyth G, Smyth C: Irritable bowel syndrome in general practice: prevalence, characteristics, and referral. *Gut* 2000, 46:78-82.

47. Österberg E, Blomquist L, Krakau I, Weinryb R, Åsberg M, Hultcrantz R: A population study on irritable bowel syndrome and mental health. *Scandinavian journal of gastroenterology* 2000, 35:264-268.

48. Boekema PJ, van Isselt EFvD, Bots ML, Smout AJ: Functional bowel symptoms in a general Dutch population and associations with common stimulants. *The Netherlands journal of medicine* 2001, 59:23-30.

49. Talley NJ, Phillips S, Melton III LJ, Wiltgen C, Zinsmeister AR: A patient questionnaire to identify bowel disease. *Annals of internal medicine* 1989, 111:671-674.

50. Mearin XB, A. Balboa, E. Baró, E. Caldwell, M. Cucala, M. Díaz-Rubio, A. Fueyo, J. Ponce, M. Roset, NJ Talley, F: Irritable bowel syndrome prevalence varies enormously depending on the employed diagnostic criteria: comparison of Rome II versus previous criteria in a general population. *Scandinavian journal of gastroenterology* 2001, 36:1155-1161.

51. Badia X, Mearin F, Balboa A, Baró E, Caldwell E, Cucala M, Díaz-Rubio M, Fueyo A, Ponce J, Roset M: Burden of illness in irritable bowel syndrome comparing Rome I and Rome II criteria. *Pharmacoeconomics* 2002, 20:749-758.

52. Baretić M, Bilić A, Jurcić D, Mihanović M, Sunić-Omejc M, Dorosulić Z, Restek-Petrović B: Epidemiology of irritable bowel syndrome in Croatia. *Collegium antropologicum* 2002, 26:85-91.

53. Bommelaer G, Dorval E, Denis P, Czernichow P, Frexinos J, Pelc A, Slama A, El Hasnaoui A: Prevalence of irritable bowel syndrome in the French population according to the Rome I criteria. *Gastroenterologie clinique et biologique* 2002, 26:1118-1123.

54. Icks A, Haastert B, Enck P, Rathmann W, Giani G: Prevalence of functional bowel disorders and related health care seeking: a population-based study. *Zeitschrift für Gastroenterologie* 2002, 40:177-183.

55. Hungin A, Whorwell P, Tack J, Mearin F: The prevalence, patterns and impact of irritable bowel syndrome: an international survey of 40 000 subjects. *Alimentary pharmacology & therapeutics* 2003, 17:643-650.

56. Bommelaer G, Poynard T, Le Pen C, Gaudin A-F, Maurel F, Priol G, Amouretti M, Frexinos J, Ruszniewski P, El Hasnaoui A: Prevalence of irritable bowel syndrome (IBS) and variability of diagnostic criteria. *Gastroentérologie clinique et biologique* 2004, 28:554-561.

57. Corazziari E, Attili A, Angeletti C, De Santis A: Biliary colic is highly prevalent, but is not the main indication for cholecystectomy, in IBS subjects with gallstones. MICOL population-based study. In *GASTROENTEROLOGY*. WB SAUNDERS CO INDEPENDENCE SQUARE WEST CURTIS CENTER, STE 300, PHILADELPHIA …; 2004: A367-A368.

58. Dapoigny M, Bellanger J, Bonaz B, des Varannes SB, Bueno L, Coffin B, Ducrotté P, Flourié B, Lémann M, Lepicard A: Irritable bowel syndrome in France: a common, debilitating and costly disorder. *European journal of gastroenterology & hepatology* 2004, 16:995-1001.

59. Hillilä M, Färkkilä M: Prevalence of irritable bowel syndrome according to different diagnostic criteria in a non‐selected adult population. *Alimentary pharmacology & therapeutics* 2004, 20:339-345.

60. Vandvik PO, Lydersen S, Farup PG: Prevalence, comorbidity and impact of irritable bowel syndrome in Norway. *Scandinavian journal of gastroenterology* 2006, 41:650-656.

61. Klooker TK, Braak B, Painter RC, De Rooij SR, Van Elburg RM, Van Den Wijngaard RM, Roseboom TJ, Boeckxstaens GE: Exposure to severe wartime conditions in early life is associated with an increased risk of irritable bowel syndrome: a population-based cohort study. *Official journal of the American College of Gastroenterology| ACG* 2009, 104:2250-2256.

62. Usai P, Manca R, Lai MA, Russo L, Boi MF, Ibba I, Giolitto G, Cuomo R: Prevalence of irritable bowel syndrome in Italian rural and urban areas. *European journal of internal medicine* 2010, 21:324-326.

63. Fosnes GS, Lydersen S, Farup PG: Constipation and diarrhoea-common adverse drug reactions? A cross sectional study in the general population. *BMC clinical pharmacology* 2011, 11:1-9.

64. Ziółkowski BA, Pacholec A, Kudlicka M, Ehrmann A, Muszyński J: Prevalence of abdominal symptoms in the Polish population. *Gastroenterology Review/Przegląd Gastroenterologiczny* 2012, 7:20-25.

65. Krogsgaard LR, Engsbro AL, Bytzer P: The epidemiology of irritable bowel syndrome in Denmark. A population-based survey in adults≤ 50 years of age. *Scandinavian journal of gastroenterology* 2013, 48:523-529.

66. Kjellström L, Molinder H, Agréus L, Nyhlin H, Talley NJ, Andreasson A: A randomly selected population sample undergoing colonoscopy: prevalence of the irritable bowel syndrome and the impact of selection factors. *European journal of gastroenterology & hepatology* 2014, 26:268-275.

67. Krogsgaard L, Engsbro A, Jones M, Bytzer P: The epidemiology of irritable bowel syndrome: Symptom development over a 3‐year period in Denmark. A prospective, population‐based cohort study. *Neurogastroenterology & Motility* 2017, 29:e12986.

68. Van den Houte K, Carbone F, Pannemans J, Corsetti M, Fischler B, Piessevaux H, Tack J: Prevalence and impact of self-reported irritable bowel symptoms in the general population. *United European gastroenterology journal* 2019, 7:307-315.

69. Schauer B, Grabe HJ, Ittermann T, Lerch MM, Weiss FU, Mönnikes H, Völzke H, Enck P, Schwille‐Kiuntke J: Irritable bowel syndrome, mental health, and quality of life: Data from a population‐based survey in Germany (SHIP‐Trend‐0). *Neurogastroenterology & Motility* 2019, 31:e13511.

70. Nakov R, Dimitrova-Yurukova D, Snegarova V, Uzunova M, Lyutakov I, Ivanova M, Madzharova K, Valkov H, Hristova R, Ivanov K: Prevalence of Irritable Bowel Syndrome, Functional Dyspepsia and their Overlap in Bulgaria: a Population-Based Study. *Journal of Gastrointestinal & Liver Diseases* 2020, 29.

71. Sperber AD, Bangdiwala SI, Drossman DA, Ghoshal UC, Simren M, Tack J, Whitehead WE, Dumitrascu DL, Fang X, Fukudo S: Worldwide prevalence and burden of functional gastrointestinal disorders, results of Rome Foundation global study. *Gastroenterology* 2021, 160:99-114. e113.

72. Brattberg G, Thorslund M, Wikman A: The prevalence of pain in a general population. The results of a postal survey in a county of Sweden. *Pain* 1989, 37:215-222.

73. Chrubasik S, Junck H, Zappe H, Stutzke O: A survey on pain complaints and health care utilization in a German population sample. *European journal of anaesthesiology* 1998, 15:397-408.

74. Catala E, Reig E, Artes M, Aliaga L, López J, Segu J: Prevalence of pain in the Spanish population: telephone survey in 5000 homes. *European journal of pain* 2002, 6:133-140.

75. Rustøen T, Wahl AK, Hanestad BR, Lerdal A, Paul S, Miaskowski C: Prevalence and characteristics of chronic pain in the general Norwegian population. *European Journal of pain* 2004, 8:555-565.

76. Breivik H, Collett B, Ventafridda V, Cohen R, Gallacher D: Survey of chronic pain in Europe: prevalence, impact on daily life, and treatment. *European journal of pain* 2006, 10:287-333.

77. Jablonska B, Soares JJ, Sundin Ö: Pain among women: associations with socio-economic and work conditions. *European Journal of Pain* 2006, 10:435-447.

78. Bouhassira D, Lantéri-Minet M, Attal N, Laurent B, Touboul C: Prevalence of chronic pain with neuropathic characteristics in the general population. *Pain* 2008, 136:380-387.

79. Landmark T, Romundstad P, Dale O, Borchgrevink PC, Kaasa S: Estimating the prevalence of chronic pain: validation of recall against longitudinal reporting (the HUNT pain study). *Pain* 2012, 153:1368-1373.

80. Azevedo LF, Costa-Pereira A, Mendonça L, Dias CC, Castro-Lopes JM: Epidemiology of chronic pain: a population-based nationwide study on its prevalence, characteristics and associated disability in Portugal. *The journal of pain* 2012, 13:773-783.

81. Björnsdóttir S, Jónsson S, Valdimarsdóttir U: Functional limitations and physical symptoms of individuals with chronic pain. *Scandinavian journal of rheumatology* 2013, 42:59-70.

82. Dueñas M, Salazar A, Ojeda B, Fernández-Palacín F, Micó JA, Torres LM, Failde I: A nationwide study of chronic pain prevalence in the general Spanish population: identifying clinical subgroups through cluster analysis. *Pain Medicine* 2015, 16:811-822.

83. Del Giorno R, Frumento P, Varrassi G, Paladini A, Coaccioli S: Assessment of chronic pain and access to pain therapy: a cross-sectional population-based study. *Journal of pain research* 2017, 10:2577.

84. Bergman S, Herrström P, Högström K, Petersson IF, Svensson B, Jacobsson LT: Chronic musculoskeletal pain, prevalence rates, and sociodemographic associations in a Swedish population study. *The Journal of rheumatology* 2001, 28:1369-1377.

85. Wijnhoven HA, De Vet HC, Picavet HSJ: Prevalence of musculoskeletal disorders is systematically higher in women than in men. *The Clinical journal of pain* 2006, 22:717-724.

86. Hagen K, Linde M, Heuch I, Stovner LJ, Zwart J-A: Increasing prevalence of chronic musculoskeletal complaints. A large 11-year follow-up in the general population (HUNT 2 and 3). *Pain Medicine* 2011, 12:1657-1666.

87. Macfarlane GJ, Beasley M, Smith BH, Jones GT, Macfarlane TV: Can large surveys conducted on highly selected populations provide valid information on the epidemiology of common health conditions? An analysis of UK Biobank data on musculoskeletal pain. *British journal of pain* 2015, 9:203-212.

88. Zondervan KT, Yudkin PL, Vessey MP, Jenkinson CP, Dawes MG, Barlow DH, Kennedy SH: Chronic pelvic pain in the community—symptoms, investigations, and diagnoses. *American journal of obstetrics and gynecology* 2001, 184:1149-1155.

89. Mohedo ED, Wärnberg J, López FB, Velasco SM, Burgos AC: Chronic pelvic pain in Spanish women: Prevalence and associated risk factors. A crosssectional study. *Clinical and Experimental Obstetrics & Gynecology* 2014, 41:243-248.

90. Margueritte F, Fritel X, Zins M, Goldberg M, Panjo H, Fauconnier A, Ringa V: The underestimated prevalence of neglected chronic pelvic pain in women, a nationwide cross-sectional study in France. *Journal of clinical medicine* 2021, 10:2481.

91. Hillman M, Wright A, Rajaratnam G, Tennant A, Chamberlain M: Prevalence of low back pain in the community: implications for service provision in Bradford, UK. *Journal of Epidemiology & Community Health* 1996, 50:347-352.

92. Smith BH, Elliott AM, Hannaford PC, Chambers WA, Smith WC: Factors related to the onset and persistence of chronic back pain in the community: results from a general population follow-up study. *Spine* 2004, 29:1032-1040.

93. Bjorck-Van Dijken C, Fjellman-Wiklund A, Hildingsson C: Low back pain, lifestyle factors and physical activity: a population based-study. *Journal of rehabilitation medicine* 2008, 40:864.

94. Heuch I, Hagen K, Heuch I, Nygaard Ø, Zwart J-A: The impact of body mass index on the prevalence of low back pain: the HUNT study. *Spine* 2010, 35:764-768.

95. Gouveia N, Rodrigues A, Eusébio M, Ramiro S, Machado P, Canhao H, Branco JC: Prevalence and social burden of active chronic low back pain in the adult Portuguese population: results from a national survey. *Rheumatology international* 2016, 36:183-197.

96. Ho KKN, Simic M, Småstuen MC, de Barros Pinheiro M, Ferreira PH, Johnsen MB, Heuch I, Grotle M, Zwart JA, Nilsen KB: The association between insomnia, c-reactive protein, and chronic low back pain: Cross-sectional analysis of the HUNT study, Norway. *Scandinavian journal of pain* 2019, 19:765-777.

97. Mäkela M, Heliövaara M, Sievers K, Impivaara O, Knekt P, Aromaa A: Prevalence, determinants, and consequences of chronic neck pain in Finland. *American journal of epidemiology* 1991, 134:1356-1367.

98. Guez M, Hildingsson C, Nilsson M, Toolanen G: The prevalence of neck pain. *Acta Orthopaedica Scandinavica* 2002, 73:455-459.

99. Guez M, Hildingsson C, Stegmayr B, Toolanen G: Chronic neck pain of traumatic and non-traumatic origin A population-based study. *Acta Orthopaedica Scandinavica* 2003, 74:576-579.

100. Leijon O, Wahlström J, Mulder M: Prevalence of self-reported neck-shoulder-arm pain and concurrent low back pain or psychological distress: time-trends in a general population, 1990–2006. *Spine* 2009, 34:1863-1868.

101. Wertli MM, Dangma TD, Müller SE, Gort LM, Klauser BS, Melzer L, Held U, Steurer J, Hasler S, Burgstaller JM: Non-cardiac chest pain patients in the emergency department: Do physicians have a plan how to diagnose and treat them? A retrospective study. *PloS one* 2019, 14:e0211615.

102. Ribas Y, Saldaña E, Martí-Ragué J, Clavé P: Prevalence and pathophysiology of functional constipation among women in Catalonia, Spain. *Diseases of the colon & rectum* 2011, 54:1560-1569.

103. Walsh K, McWilliams S, Maher M, Quigley EM: The spectrum of functional gastrointestinal disorders in a tertiary referral clinic in Ireland. *Irish journal of medical science* 2012, 181:81-86.

104. Rey E, Balboa A, Mearin F: Chronic constipation, irritable bowel syndrome with constipation and constipation with pain/discomfort: similarities and differences. *Official journal of the American College of Gastroenterology| ACG* 2014, 109:876-884.

105. Enck P, Leinert J, Smid M, Köhler T, Schwille-Kiuntke J: Functional constipation and constipation-predominant irritable bowel syndrome in the general population: data from the GECCO study. *Gastroenterology Research and Practice* 2016, 2016.

106. Pannemans J, Van den Houte K, Fischler B, Piessevaux H, Carbone F, Tack J: Prevalence and impact of self‐reported painful and non‐painful constipation in the general population. *Neurogastroenterology & Motility* 2020, 32:e13783.

107. Breckan RK, Asfeldt AM, Straume B, Florholmen J, Paulssen EJ: Prevalence, comorbidity, and risk factors for functional bowel symptoms: a population-based survey in Northern Norway. *Scandinavian journal of gastroenterology* 2012, 47:1274-1282.

108. Dantoft TM, Nordin S, Andersson L, Petersen MW, Skovbjerg S, Jørgensen T: Multiple chemical sensitivity described in the Danish general population: Cohort characteristics and the importance of screening for functional somatic syndrome comorbidity—The DanFunD study. *PloS one* 2021, 16:e0246461.

109. Wenzel HG, Mykletun A, Nilsen TIL: Symptom profile of persons self-reporting whiplash: a Norwegian population-based study (HUNT 2). *European Spine Journal* 2009, 18:1363-1370.

110. Olafsdottir L, Gudjonsson H, Jonsdottir H, Thjodleifsson B: Stability of the irritable bowel syndrome and subgroups as measured by three diagnostic criteria–a 10‐year follow‐up study. *Alimentary pharmacology & therapeutics* 2010, 32:670-680.

111. Zagari RM, Law GR, Fuccio L, Cennamo V, Gilthorpe MS, Forman D, Bazzoli F: Epidemiology of functional dyspepsia and subgroups in the Italian general population: an endoscopic study. *Gastroenterology* 2010, 138:1302-1311.

112. Ebling B, Jurcic D, Barac KM, Bilic A, Bajic I, Martinac M, Pribic S, Vcev A: Influence of various factors on functional dyspepsia. *Wiener klinische Wochenschrift* 2016, 128:34-41.

113. Maaranen P, Tanskanen A, Hintikka J, Honkalampi K, Haatainen K, Koivumaa-Honkanen H, Viinamäki H: The course of dissociation in the general population: A 3-year follow-up study. *Comprehensive Psychiatry* 2008, 49:269-274.
